# Structural and functional insights into lysine acetylation of cytochrome *c* using mimetic point mutants

**DOI:** 10.1002/2211-5463.13284

**Published:** 2021-11-09

**Authors:** Inmaculada Márquez, Gonzalo Pérez‐Mejías, Alejandra Guerra‐Castellano, José Luis Olloqui‐Sariego, Rafael Andreu, Juan José Calvente, Miguel A. De la Rosa, Irene Díaz‐Moreno

**Affiliations:** ^1^ Institute for Chemical Research (IIQ) Scientific Research Centre Isla de la Cartuja (cicCartuja) University of Seville – CSIC Spain; ^2^ Departament of Physical Chemistry University of Seville Spain

**Keywords:** acetylation, bioenergetics, cytochrome *c*, electron transport chain, post‐translational modifications

## Abstract

Post‐translational modifications frequently modulate protein functions. Lysine acetylation in particular plays a key role in interactions between respiratory cytochrome *c* and its metabolic partners. To date, *in vivo* acetylation of lysines at positions 8 and 53 has specifically been identified in mammalian cytochrome *c*, but little is known about the structural basis of acetylation‐induced functional changes. Here, we independently replaced these two residues in recombinant human cytochrome *c* with glutamine to mimic lysine acetylation and then characterized the structure and function of the resulting K8Q and K53Q mutants. We found that the physicochemical features were mostly unchanged in the two acetyl‐mimetic mutants, but their thermal stability was significantly altered. NMR chemical shift perturbations of the backbone amide resonances revealed local structural changes, and the thermodynamics and kinetics of electron transfer in mutants immobilized on gold electrodes showed an increase in both protein dynamics and solvent involvement in the redox process. We also observed that the K8Q (but not the K53Q) mutation slightly increased the binding affinity of cytochrome *c* to its physiological electron donor, cytochrome *c*_1_—which is a component of mitochondrial complex III, or cytochrome *bc*_1_—thus suggesting that Lys8 (but not Lys53) is located in the interaction area. Finally, the K8Q and K53Q mutants exhibited reduced efficiency as electron donors to complex IV, or cytochrome *c* oxidase.

## Graphical abstract

Acetylation is the main post‐translational modification of proteins in eukaryotic cells, affecting more than 60% of mitochondrial proteins. Mammalian cytochrome *c*, in particular, is found acetylated at lysines 8 and 53. Here, we characterize two acetyl‐mimetic mutants (Lys‐to‐Gln mutation) of human cytochrome *c* to gain insight into the structural and functional changes that could further explain the acetylation role in cancer and other diseases.

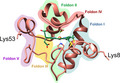

**Abbreviations**
A: pre-exponential factor
AcK: acetyl-lysine
ANOVA: one-way analysis of variance
BLI: Bio-layer interferometry
C*c*
: cytochrome *c*
C*c*_1_
: cytochrome *c*_1_
C*c*O: cytochrome *c* oxidase
CIII: complex III
CIV: complex IV
CSP: chemical shift perturbation
DMEM: Dulbecco’s modified Eagle’s medium
*E. coli*
: *Escherichia coli*
*E* ^0^
: midpoint reduction potential
*E*_1/2_
: midpoint redox potential value
ETC: electron transport chain
HSQC: heteronuclear single quantum correlation
ITC: isothermal titration calorimetry
*K*_D_
: dissociation equilibrium constant
*k*_s_
: electron transfer rate constant
LB: luria-Bertani
MALDI-TOF: matrix-assisted laser desorption/ionization-time of flight
MD: molecular dynamics
MEF: mouse embryonic fibroblast
MOA: 8-Mercaptooctanoic acid
MOOL: 8-Mercapto-1-octanol
NHE: normal hydrogen electrode
NOESY: nuclear Overhauser effect spectroscopy
P1: first principal component
PCA: principal component analysis
PDB: protein data bank
PMSF: phenylmethanesulfonyl fluoride
PTMs: post-translational modifications
RG: radius of gyration
RMSF: root mean square fluctuation
RSA: rabbit serum albumin
SAMs: self-assembled monolayers
SDS/PAGE: sodium dodecyl sulfate polyacrylamide gel electrophoresis
*T*
: temperature
*T*_m_
: midpoint melting temperature
TOCSY: total correlation spectroscopy
UV: ultraviolet
Vis: visible
WT: wild-type
ε: extinction coefficient

Post‐translational modifications (PTMs) have traditionally been associated with mechanisms that cells use to regulate and expand the functionality of their biomolecules [1, 2, 3]. These modifications can be reversible or irreversible and can include covalent addition of complex molecules, chemical groups, and proteins/peptides, as well as protein cleavage and amino acid modification. Recent studies however have shown that certain PTMs (including acetylation, glycosylation, methylation, nitration, S‐palmitoylation, phosphorylation, S‐nitrosylation, sumoylation, and ubiquitination) or the loss of modification sites in proteins are responsible for the development of certain diseases [4, 5, 6, 7]. Based on the PTM database (PTMD), phosphorylation is the most frequent PTM associated with human disease (http://ptmd.biocuckoo.org/) [6]. These data are striking because acetylation is the major covalent modification to occur in eukaryotic cells, where over 60% of mitochondrial proteins (soluble and membrane‐embedded) have one or more acetylation sites [8, 9, 10, 11]. Acetylation actually regulates diverse protein properties, including DNA interaction, subcellular localization, transcriptional activity, and stability [12]. Acetylation is a reversible enzymatic process carried out by lysine acetyltransferases, in which the ε‐amino group of lysine residues is reversibly modified by the acetyl group. Interestingly, the acetylation process can take place by means of a non‐enzymatic mechanism in the mitochondrial matrix, where the addition of the acetyl group is promoted by high concentrations of acetyl‐CoA and the alkaline pH [13].

A principal mitochondrial protein affected by PTMs is respiratory cytochrome *c* (C*c*), which has also been described as a target for lysine acetylation [14]. Under homeostasis, C*c* is known to be located in the mitochondrial intermembrane space and acts as an electron carrier between complex III (CIII) and complex IV (CIV) in the electron transport chain (ETC). It was recently reported that CIII and CIV host two C*c*‐binding sites each: the so‐called *proximal*, or catalytic site, which is the relevant functional position for electron transfer, and the *distal*, or non‐productive site, which facilitates the turnover and sliding of C*c* molecules [15, 16]. In the case of the respiratory supercomplexes, the close arrangement of CIII and CIV suggests a ‘restrained diffusion pathway’ for C*c* molecules between the two complexes [17]. Nevertheless, several internal and external cell stimuli can induce the translocation of C*c* to other cell compartments (nucleus, vacuoles, zymogen granules, cytosol, and even the rough endoplasmic reticulum), where C*c* behaves as a moonlighting protein able to interact with an ample set of non‐redox proteins [18, 19, 20, 21, 22, 23, 24, 25, 26, 27, 28].

As mentioned above, C*c* can be acetylated on its lysine residues [29]. Several studies with chemically acetylated hemeprotein showed that lysine acetylation modulates respiratory chain functioning by inducing a substantial drop in the C*c* reduction/oxidation rate [29] and, consequently, a decrease in C*c*‐mediated electron transfer [30]. Notably, these *in vitro* acetylation assays simultaneously affect multiple lysines, being difficult to assign any acetylation effect to any particular lysine. Downregulation of mitochondrial electron flow promotes inhibition of cellular respiration, also known as the Warburg effect [31]. So far, *in vivo* acetylation of both Lys8 and Lys53 has only been described in mammalian C*c*. Acetylation of Lys8 was observed in a proteomic analysis of mouse liver mitochondria, whereas acetylation of Lys53 was identified in prostatic cancer cells [8, 32]. This association with prostate cancer led to acetylation of Lys53 being the first to become the object of study [32].

The interaction between C*c* and its partners depends strongly on the ionic strength of the media, revealing the importance of charged residues such as lysines in its associations [33]. Lys8 and Lys53, with their side chains exposed to the solvent, are involved in the binding of C*c* to most of its metabolic partners as they contribute to a positively charged Lys‐rich patch at the hemeprotein surface [16, 22, 34, 35, 36, 37]. Since acetylation neutralizes the positive charge of lysines, this PTM might induce drastic changes in the physicochemical and structural features of C*c* and could therefore alter interactions with its metabolic partners.

The difficulty of preserving the acetylation state of C*c* and the low yield obtained from cell extracts make it challenging to analyze. Furthermore, the specific acetyltransferase acting on the hemeprotein remains unknown. Hence, recourse was made to amino acid point mutations to mimic the PTM. The Lys‐to‐Gln mutation can mimic the acetylation‐dependent neutralization of the positive lysine charge, so that the resulting C*c* species could be used as acetyl‐mimetic mutants. As part of a strategy to gain insight into the structural and functional analysis of lysine‐acetylated C*c*, we here characterize two acetyl‐mimetic mutants of C*c*, namely K8Q and K53Q C*c*, using K8A and K53A mutants as controls.

## Methods

### Site‐directed mutagenesis, protein expression, and purification of recombinant proteins

Site‐directed mutagenesis was performed on a pBTR1 plasmid comprising the CYCS gene coding for human C*c*, along with the CYC3 gene of the yeast C*c* heme lyase [38]. The CYC3 gene is required for the proper cytoplasmic maturation of C*c*, as the heme lyase covalently attaches the heme group to the apoprotein of C*c*. Primers used for mutagenic PCR are detailed in Table S1.

The expression of wild‐type (WT) and acetyl‐mimetic C*c* species was performed as previously described [39]. Briefly, *Escherichia coli* (*E. coli*) BL21 (DE3) cells were transformed with either pBTR1‐C*c* WT or pBTR1‐C*c* acetyl‐mimetic plasmids. The cells were cultured at 30 °C with agitation (150 rpm) in Luria‐Bertani (LB) medium supplemented with 100 μg·mL^−1^ ampicillin for 20 h. Cells were harvested by centrifugation (9000 ***g*** for 10 min), suspended in lysis buffer (10 mm Tricine‐NaOH, pH 8.5, supplemented with 1 mm phenylmethylsulfonyl fluoride (PMSF), 0.02 mg·mL^−1^ DNase and complete protease inhibitor) and physically ruptured by sonication. Cellular debris was separated by centrifugation at 14 000 ***g*** for 30 min at 4 °C. For the NMR experiments, ^15^N‐labeled protein was expressed in minimal M9 medium supplemented with 16.7 μg·mL^−1^ δ‐aminolevulinic acid hydrochloride and ^15^NH_4_Cl as sole nitrogen source. Protein purification was carried out by ion chromatography in a Nuvia‐S (Bio‐Rad, Hercules, CA, USA) column using a FPLC system (Bio‐Rad). The purity of protein samples was routinely tested by electrophoretic (Fig. S1A) and spectrophotometric analyses (see below). In all cases, the A_280_/A_550_ ratio of the resulting C*c* preparations in the reduced state was about 1.1—as previously reported to be in pure C*c* samples [39]—with a maximum 10% deviation. Immunoblot was performed with rabbit anti‐human C*c* serum (obtained by immunizing male rabbits with full‐length recombinant C*c* suspended in a 0.85% NaCl solution) as primary antibody. HRP‐conjugated secondary antibody was used for detection (Sigma‐Aldrich Co., St. Louis, MO, USA, catalog number A0545). The immunoreactive bands were detected using Amersham ECL Plus Western Blotting Detection Reagents (GE Healthcare Life Sciences, Chalfont St. Giles, UK). The molecular masses of C*c* variants were checked by running a sodium dodecyl sulfate–polyacrylamide gel electrophoresis (SDS/PAGE) and matrix‐assisted laser desorption/ionization‐time of flight (MALDI‐TOF). Tryptic digestion analysis confirmed the point mutations of the C*c* species. Protein concentration was determined by visible (Vis) spectrophotometry, using an extinction coefficient (ε_550nm_) of 28.92 mm ^−1^·cm^−1^ for reduced C*c* [39]. Protein samples were dialyzed against 10 mm sodium phosphate buffer pH 5.5 or pH 7.4 for NMR, or CD and bio‐layer interferometry (BLI) experiments.

The C10A mutant of *Arabipdosis thaliana* cytochrome *c*_1_ (C*c*_1_), used to avoid protein dimerization, was expressed as previously described [15]. *E. coli* BL21 (DE3) cells were co‐transformed with pET‐C*c*_1_‐C10A and pEC86—coding for the CYC3 gene of the yeast C*c* heme lyase—to produce the soluble domain of C*c*_1_. Cells were grown for 24 h at 150 rpm and 30 °C in LB‐rich medium supplemented with 16.7 μg·mL^−1^ δ‐aminolevulinic acid hydrochloride, 50 μg·mL^−1^ kanamycin, and 12 μg·mL^−1^ chloramphenicol. Cells were collected by centrifugation (9000 ***g*** for 10 min) and then suspended in lysis buffer (20 mm Tris/HCl, pH 8.0, supplemented with 1 mm PMSF, 0.02 mg·mL^−1^ DNase and complete protease inhibitor). The periplasmic fraction was obtained by the freeze‐thaw lysis method. The resulting suspension was centrifuged at 20 000 ***g*** for 30 min and loaded onto a Nuvia‐Q column (Bio‐Rad). C*c*_1_ was eluted with a NaCl linear gradient in 20 mm Tris/HCl, pH 8.0. The fractions containing C*c*_1_ were further purified by FPLC using a gel filtration ENrich SEC 70 column (Bio‐Rad). Protein concentration was determined by Vis spectrophotometry, using an extinction coefficient (ε_552nm_) of 28 mm ^−1^·cm^−1^ for reduced C*c*_1_ [15]. The recombinant protein was dialyzed against 10 mm sodium phosphate buffer pH 7.4 for BLI experiments.

### CD spectroscopy

CD spectra were recorded using a Jasco® J‐815 spectropolarimeter equipped with a Peltier temperature control system. The secondary structure analyses were carried out by recording far‐ultraviolet (UV) CD spectra (185–250 nm) at 25 °C in a 1‐mm quartz cuvette. Samples contained 3 μm protein in 10 mm sodium phosphate (pH 7.4), supplemented with 10 μm potassium ferricyanide. 20 scans were averaged for each sample.

The coordination of the heme iron atom to the *S*_δ_ atom of Met80 (the sixth axial ligand of heme group) was analyzed by visible (B‐band) recording near‐UV/Vis CD spectra (300–600 nm) in a 10 mm quartz cuvette at 25 °C. Samples contained 30 μm protein in 10 mm sodium phosphate (pH 7.4), supplemented with 100 μm potassium ferricyanide.

Thermal unfolding was monitored between 10 °C and 105 °C (with a heating rate of 1 °C·min^−1^) by recording the CD signal at far‐UV in a 10 mm quartz cuvette. For these assays, the oxidized C*c* species were at 3 μm final concentration in 10 mm sodium phosphate (pH 7.4), supplemented with 10 μm potassium ferricyanide. The heme coordination of the C*c* species was monitored from 10 °C and 105 °C (with a heating rate of 1 °C·min^−1^) by recording near‐UV/Vis CD spectra from solutions in a 10 mm quartz cuvette. Samples contained 30 μm C*c* in 10 mm sodium phosphate buffer (pH 7.4), supplemented with 100 μm potassium ferricyanide. The midpoint melting temperature (*T*_m_) values were obtained from the fits of principal components obtained from CD and fluorescence spectra measured on temperature ramps.

### Electronic absorption spectroscopy

The redox state reversibility of heme group was checked by recording absorption spectra from 350 to 600 nm after the addition of 10 equivalents of potassium ferricyanide as an oxidizing agent and 50 equivalents of sodium ascorbate as a reducing agent. Spectra of 5 µm of C*c* species were recorded at 25 °C in 10 mm sodium phosphate buffer (pH 7.4).

The coordination of the heme group of C*c* was analyzed by monitoring the absorption changes at 699 nm, indicative of the heme Fe‐Met80(S_δ_) bond as reported previously [40]. Electronic absorption spectra were recorded in the 600–750 nm range at 25 °C, using a Jasco® V‐650 spectrophotometer in a 1‐mL quartz cuvette with a path length of 10 mm. Samples contained 0.2 mm oxidized C*c* in 10 mm sodium phosphate (pH 5.8), supplemented with 0.2 mm potassium ferricyanide. For pH titration studies, the pH of each sample was adjusted to the reported values by adding aliquots of 0.1–0.5 M NaOH or 0.1–0.5 M HCl. To calculate the p*K*_a_ value of each protein, the absorbance changes at 699 nm were fitted to the Henderson‐Hasselbalch equation expressed in the following terms [41]:

(1) $$Y={\text{Abs}}_{\min}+\frac{{\text{Abs}}_{\max}‐{\text{Abs}}_{\min}}{1+e^{(X‐pKa)/\text{NS}}}$$

where Y is the experimental value for absorbance at 699 nm, Abs_max_ and Abs_min_ are the maximum and minimum values for absorbance at 699 nm, X is the pH of the protein sample, p*K*_a_ is the apparent p*K*_a_ value for alkaline transition, and NS is the slope of the sigmoid.

### Molecular dynamics simulations

The initial structures were based on the NMR structure of reduced human C*c* (Protein Data Bank, PDB, ID: 2N9I; [42]). All reduced mutants (K8A, K8Q, K8AcK, K53A, K53Q, and K53AcK) structural models were built using the WT protein as a template with UCSF Chimera software [43]. The force field parameters for acetyl‐lysine [44] and heme group [45] were used to generate the topology and coordinate files, required for the simulation, with TLEAP module of AMBER [46].

Molecular dynamic (MD) simulations were performed using the OpenMM [47] software in CUDA platform with AMBER 14SB force field [48] and particle mesh Ewald electrostatics with an Ewald summation cut‐off of 10 Å. For each simulation, the system was neutralized with chlorine counter‐ions according to the total charge of the protein and solvated with optimal‐3‐charge, 4‐point rigid water model water molecule [49]. The whole system was subjected to 2500 steps of energy minimization at 298 K. Langevin thermostat was used to control the temperature with a friction coefficient of 1 ps^−1^ and a step size of 0.002 ps. Each system was subjected to 900 ns MD simulation. For the trajectory analysis, the CPPTRAJ module of AMBER was used [50] to analyze the last 50 ns of simulation.

### NMR spectroscopy

NMR experiments were performed on a 700 MHz Bruker Avance‐III spectrometer equipped with a cryoprobe. Backbone amide group resonances of C*c* variants were monitored by recording 2D ^15^N‐^1^H heteronuclear single quantum correlation (HSQC) experiments. Samples contained 150 μm of reduced ^15^N‐labeled C*c* in 20 mm citrate buffer (pH 5.5), 5% D_2_O and sodium ascorbate to ensure the redox state of the sample. NMR assignments of the ^15^N and ^1^H nuclei of reduced WT C*c* (BMRB accession number 25907) were taken from previous work [42]. Additional 3D ^15^N‐^1^H total correlation spectroscopy (TOCSY)‐HSQC and 3D ^15^N‐^1^H nuclear Overhauser effect spectroscopy (NOESY)‐HSQC experiments were recorded to identify the resonances of the Lys‐Gln or Lys‐Ala C*c* mutants. The experimental data were processed using Bruker TopSpin NMR 4.1.1 software (Bruker), and chemical shift perturbations (CSPs) were analyzed with NMRFAM‐Sparky NMR assignment tool [51].

### Bio‐layer interferometry measurements

BLI measurements were performed on the Octet RED 96e platform (FortéBio) using Ni‐NTA (NTA) Biosensors (FortéBio). The protocol used for the kinetic analysis is detailed in Table S2.

The biosensors were hydrated in a 96‐well black plate for 10 min with BLI buffer containing 10 mm sodium phosphate (pH 7.4) supplemented with 0.01 μm rabbit serum albumin (RSA) and 0.01% Triton X‐100 as blocking agents to reduce non‐specific binding to the biosensor. The biosensors were incubated with 200 μL of His_6_‐tagged C*c*_1_ sample (50 μg·mL^−1^ diluted in BLI buffer) at the loading step for immobilization of the analyte into the NTA matrix. Binding of WT and acetyl‐mimetic C*c* variants was analyzed at ligand concentrations between 0.3215 and 10 μm diluted in BLI buffer and performed in duplicate. The surface of the biosensor was regenerated by 10 mm glycine (pH 1.7) using the standard protocol described by the manufacturer. The experimental data were processed with Data Analysis HT 12.0 software (fortébio) and analyzed with Origin Lab Origin(Pro), Version 2019 (OriginLab Corporation, Northampton, MA, USA), using the non‐linear equation described by Müller‐Esparza *et al*. [52]:

(2) $$Y=\frac{B_{\max}\cdot X}{K_{D}+X}+\text{NS}\cdot X$$

where Y is the wavelength shift at equilibrium, B_max_ is the maximum wavelength shift, X is the effector complex concentration, *K*_D_ is the equilibrium dissociation constant, and NS is the slope of the linear components.

### Isolation of mitochondria from mouse embryonic fibroblast cell lines

Mouse embryonic fibroblast (MEF) cell lines were cultured at 37 °C in a humidified atmosphere of 5% CO_2_. MEF cells were cultured in Dulbecco’s modified Eagle’s medium (DMEM; Sigma‐Aldrich Co.) containing 4500 mg·L^−1^ glucose (Sigma‐Aldrich Co.), supplemented with 10% heat‐inactivated fetal bovine serum (Sigma‐Aldrich Co.), 2 mm L‐glutamine, 100 U·mL^−1^ streptomycin, 100 μg·mL^−1^ penicillin and 0.11 mg·mL^−1^ sodium pyruvate (Sigma‐Aldrich Co.). Cells were grown to 80% confluence in 24‐well plates with 500 μL of DMEM. Mitochondria were obtained by differential centrifugation as reported previously [53].

### Cytochrome c oxidase activity

To assess the functionality of acetyl mimetics as electron donors, we tested their ability to reduce cytochrome *c* oxidase (C*c*O) in a mitochondrial context using a C*c*O activity kit (Biochain, Newark, CA, USA), as reported previously [54]. Activity was measured on mitochondria isolated from MEF cell lines in a Jasco® V‐650 spectrophotometer in a 0.1‐mL quartz cuvette with 10 mm path length, according to the manufacturer’s instructions. The Abs_550_ slopes of at least three independent experiments were calculated and correlated with C*c*O activity values.

### Statistical analysis

Data were analyzed using Origin Lab Origin(Pro), Version 2019 (OriginLab Corporation, Northampton, MA, USA). Data are expressed as means ± SD. One‐way analysis of variance (ANOVA) was employed for comparisons of multiple groups.

### Electrochemical measurements

Linear scan voltammetric measurements were performed with an AUTOLAB PGSTAT 30, from Eco Chemie B.V, in a three‐electrode undivided glass cell, equipped with a gas inlet and thermostatted with a water jacket. Working electrodes were polycrystalline gold with a 0.0314 cm^2^ geometric area. Prior to performing measurements, the gold surface was cleaned by successively polishing it with 0.3 and 0.05 µm alumina, rinsed with Millipore water, then sonicated in absolute ethanol to remove residual alumina. Then, the electrode surface was dried and chemically cleaned using ‘piranha’ solution (7 : 3 mixture of concentrated H_2_SO_4_ (95%) and 30% v/v H_2_O_2_). Gold electrodes were modified with thiol self‐assembled monolayers (SAMs) by immersing the electrode in ethanol solutions containing 1 mm 8‐mercaptooctanoic acid (MOA) and 2.5 mm 8‐mercapto‐1‐octanol (MOOL), from Sigma‐Aldrich Co., for 30 min at room temperature. Protein immobilization was carried out by depositing on the modified electrode a 15 µL drop of a solution containing 20 µm protein, 10 mm sodium phosphate buffer solution (pH 7) for 45 min at 4 °C. After protein incubation, the electrodes were rinsed with water and washed with the working buffer solution. The counter and reference electrodes were a Pt bar and an Ag/AgCl/NaCl‐saturated electrode, respectively. Reported potential values were corrected to the normal hydrogen electrode (NHE) potential scale by adding +192 mV to the experimental potential values. Working solutions contained 20 mm sodium phosphate buffer as supporting electrolyte, at pH 7.0 ± 0.1. All measurements were performed under argon atmosphere.

## Results and Discussion

### Physicochemical features of cytochrome c are mainly unchanged in the two acetyl‐mimetic mutants

**Fig. 1:**
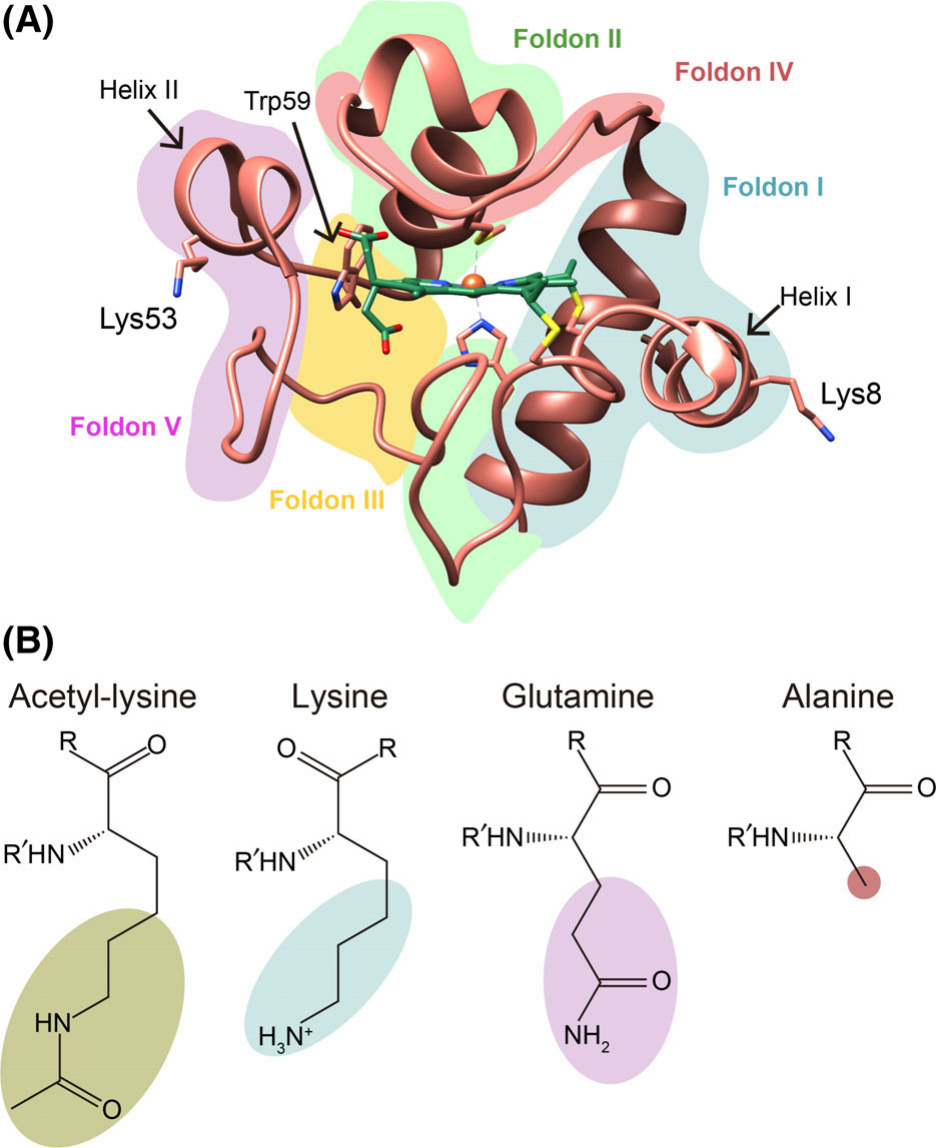
3D structure of cytochrome *c* and formula of selected amino acids. (A) Ribbon representation of human C*c* structure (PDB, ID: 2N9I [42]), with the heme group (green color) and Lys8, Lys53, and Trp59 represented by sticks. The different foldon units are colored as follows: foldon I in blue, foldon II in green, foldon III in yellow, foldon IV in red, and foldon V in purple. (B) Chemical structure of acetyl‐lysine, lysine, glutamine, and alanine.

C*c* contains 18 lysine residues but only Lys8 and Lys53 are acetylated *in vivo* [8, 32]. Each residue is located in opposite regions of the hemeprotein (Fig. 1A): Lys8 in helix I and Lys53 in helix II, with their respective side chains well exposed to the solvent. To explore how acetylation of Lys8 and Lys53 influences the physicochemical features of C*c*, we designed two acetyl‐mimetic mutants of C*c*, replacing the corresponding lysines with glutamines (K8Q and K53Q C*c* mutants) to resemble the change in charge that occurs when lysine becomes acetylated (Fig. 1B). To check the specific effect of these point mutations, Lys‐to‐Ala mutants were also studied (K8A and K53A, Fig. 1B).

**Fig. 2:**
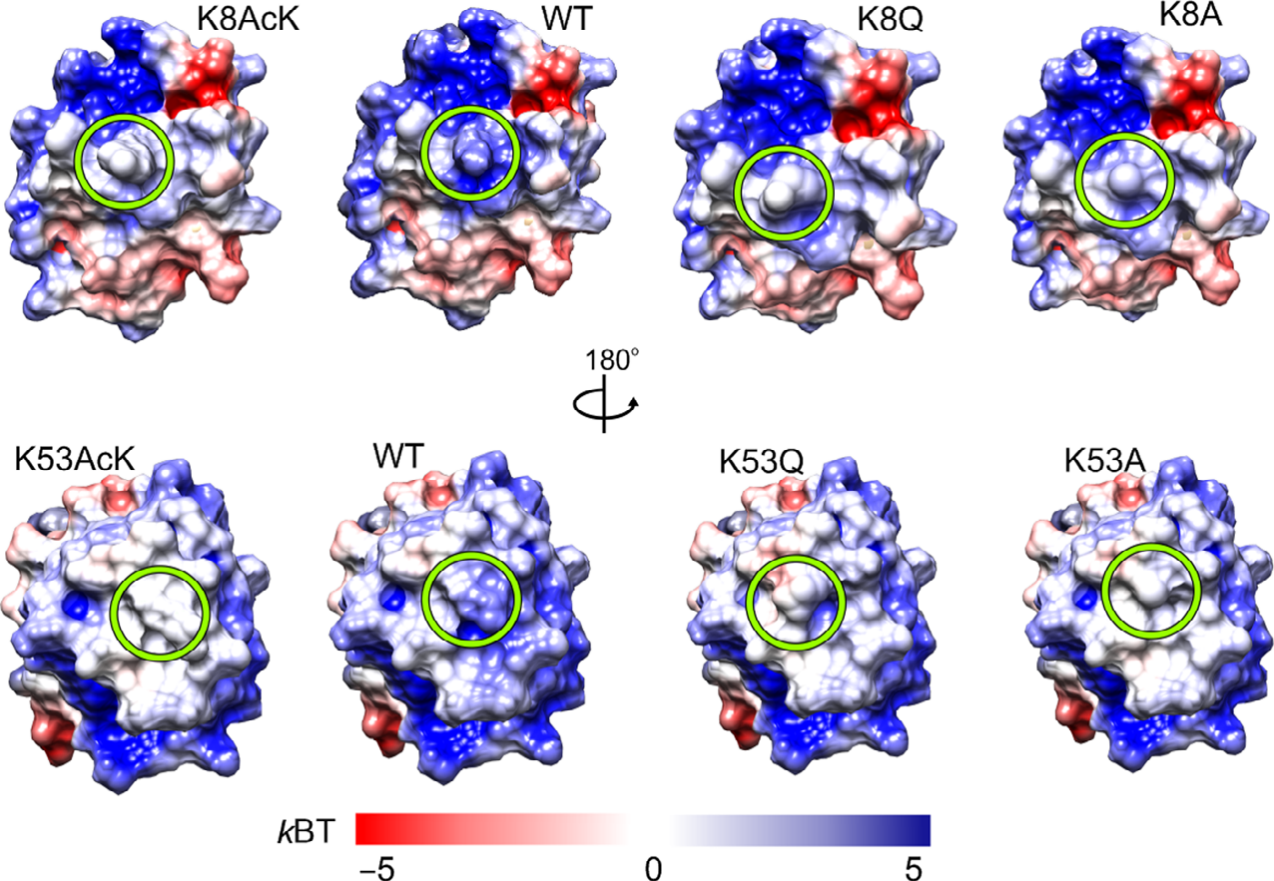
Surface electrostatic potentials of WT and mutant species of cytochrome *c*. Electrostatic potentials were calculated at 150 mm ionic strength. Green circles denote mutated residues at position 8 (*upper*) and 53 (*lower*).

Since mutation of lysine to glutamine or alanine neutralizes the positive charge, we first calculated the surface electrostatic potential of the WT, point mutants (K8A, K8Q, K53A, and K53Q), and acetylated forms (K8AcK and K53AcK) of C*c* at physiological ionic strength. In all cases, the lysine substitution resulted in a loss of positive potential at the surface area surrounding the mutated residue (Fig. 2), consistent with the charge difference between the ionic amino‐terminal group of lysine in WT C*c* and the neutral character of acetyl‐lysine, glutamine, or alanine in the mutants. The similar surface electrostatic potentials of the acetyl‐lysine variants (K8AcK and K53AcK) and the glutamine mutants (K8Q and K53Q) suggest that the Lys‐to‐Gln substitution could lead to good mimetics of acetylated C*c*.

**Fig. 3:**
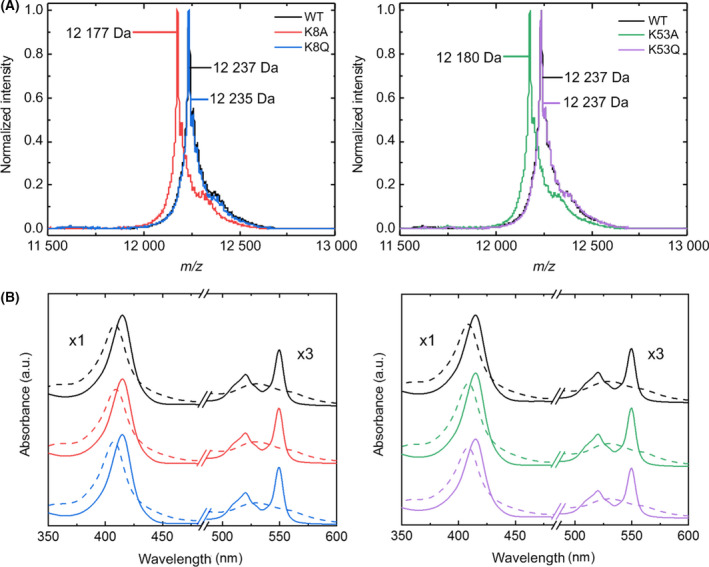
Mass and visible absorption spectra of WT and mutant species of cytochrome *c*. (A) MALDI‐TOF spectra of WT and C*c* mutants. The molecular mass of WT, K8Q, and K53Q C*c* is 12 235 Da, whereas that of K8A and K53A C*c* is 12 179 Da. (B) Visible absorption spectra of oxidized (dashed line) and reduced (solid line) C*c* in the presence of potassium ferricyanide and sodium ascorbate, respectively. The color code is the same as in panel (A). a.u., arbitrary units.

Glutamine and alanine site‐directed mutants were thus designed, expressed, and purified (see Methods section). The molecular mass of all purified mutants was corroborated by SDS/PAGE (Fig. S1A) and MALDI‐TOF mass spectrometry (Fig. 3A). The peak closest to 12 500 m*/z* in all mass spectra corresponds to the formation of a Schiff base adduct, as described previously [55]. Since the molecular masses of the glutamine mutants (K8Q and K53Q) were the same as that of the WT C*c*, protein spots from SDS/PAGE bands were subjected to trypsin digestion to confirm the Lys‐to‐Gln substitution from the masses of the resulting fragments (Fig. S1B). UV‐Vis absorption spectra clearly confirmed the typical hallmarks of *c*‐type cytochromes in all mutants (Fig. 3B). The spectra for WT and mutant C*c* in the reduced state show a sharp maximum at ca. 415 nm (Soret band), as well as two lower maximums at 520 and 550 nm (α and β bands, respectively) that merge in the oxidized state. The redox state reversibility of the heme group in all samples was checked by recovering original absorption spectra upon addition of potassium ferricyanide or sodium ascorbate as oxidizing or reducing agent, respectively.

**Fig. 4:**
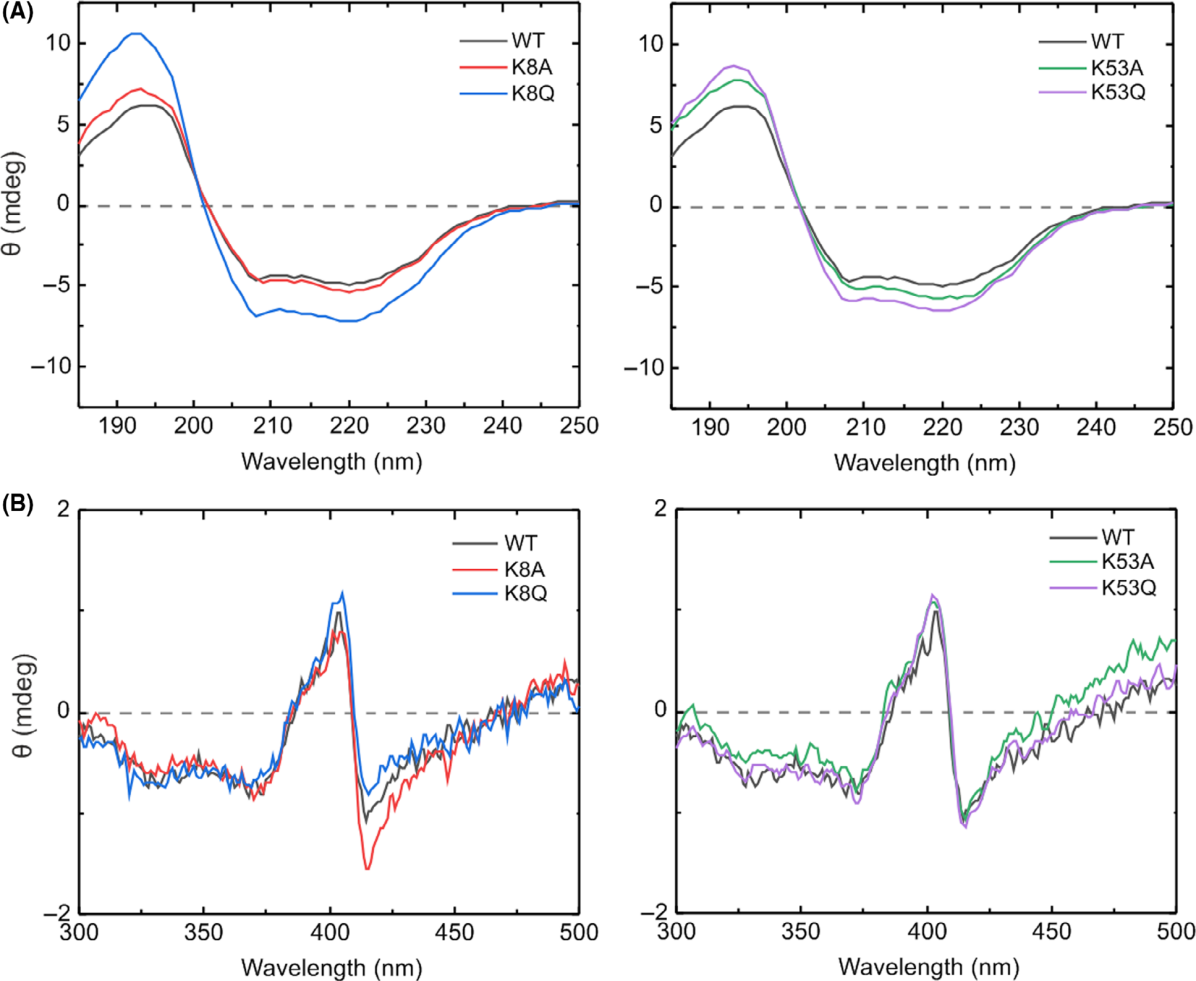
Analysis of the secondary structure and heme environment of the WT and mutant species of cytochrome *c*. (A) Far‐UV and (B) near‐UV/Vis CD spectra of oxidized WT, K8A, K8Q, K53A, and K53Q C*c* at 3 and 30 µm, respectively.

The effect of mutations on the secondary structure of C*c* was analyzed by far‐UV CD spectroscopy of WT and mutant C*c* species. The most prominent change was observed in the 195‐nm band with the K8Q mutant, suggesting a substantial increase in α‐helix content (Fig. 4). A detailed analysis using the cdpro software package (Fig. S2) not only confirmed the increase in α‐helix content in K8Q but also revealed a significant increase in K53Q.

Since subtle changes in the heme group can alter the redox properties and physiological functions of C*c*, the CD spectra in the visible region were also recorded to provide further information on the heme environment [56]. In its native form, the heme iron atom of C*c* is hexacoordinated, with the fifth and sixth axial ligands being His18 and Met80, respectively [57]. The CD spectra of WT C*c* and all mutants clearly exhibited the distinctive B‐band splitting corresponding to the Cotton effect in the Soret absorption region [58]. Taken together, these findings confirm that Lys8 and Lys53 mutations have a negligible effect on the heme environment.

Since the stability of the heme crevice is preserved under physiological conditions, the effect of pH on the heme environment was checked by Vis absorption spectroscopy. In the pH range of 1–12, at least five different conformations of C*c* have been described as a result of changes in protein folding and/or heme axial coordination [59]. The transition from state III to state IV, called *alkaline transition*, is induced by an increase in pH [60]. This state transformation involves replacing the axial ligand Met80 with a lysine residue (Lys72, Lys73, or Lys79) [61, 62]. The interaction between heme iron and Met80 in the oxidized form of C*c* causes a maximum charge‐transfer peak in the absorption spectra at 699 nm [40, 63], which disappears as the alkaline transition occurs (Fig. S3, *left* panels). The presence of the 699 nm maximum at the initial pH value (pH ca. 6.3) indicates the integrity of protein folding [64]. The intensity of this charge‐transfer peak was recorded in the pH 6.0–12.5 range, and the fitting of titration curves (Fig. S3, *right* panels) provided the p*K*_a_ values for the alkaline transition of all mutants included in this study. Considering that Tyr48‐phosphomimetic C*c* mutants have lower but biologically relevant p*K*_a_ transition values [40, 65], one would expect that the alkaline transition of mutants at Lys53, which is in the same loop as Tyr48, would also be altered. However, the p*K*_a_ values of all mutants were very close to that of WT C*c* (Table 1). These results suggest that the native heme environment is preserved in acetyl‐mimetic C*c* species, consistent with exposure of the side chains of lysine, alanine and glutamine residues at positions 8 and 53 to solvent without altering the hydrogen bond pattern around the heme crevice [40]. Conservation of Met80‐Fe bond distance, as predicted by theoretical MD calculations, also confirms the preservation of heme moiety (Fig. S4).

**Table 1:** p*K*_a_ values for alkaline transition in oxidized WT and mutant cytochrome *c*.

	p*K*_a_
WT	9.48 ± 0.05
K8A	9.54 ± 0.03
K8Q	9.56 ± 0.06
K53A	9.42 ± 0.04
K53Q	9.31 ± 0.03

### Thermal stability of cytochrome c is differentially modified depending on the mutated residue

The conformational stability of C*c* depends greatly on the protein environment, as well as the residues surrounding the heme group [66]. Changes in H‐bond networks, hydrophobic interactions, steric restrictions, and even the protein sequence can alter the *T*_m_ of the protein. To explore the effect of temperature on the secondary structure and heme moiety of C*c* species, thermal stability assays were monitored by recording the CD and fluorescence spectra in the far‐UV and near‐UV/Vis ranges, respectively, in the temperature interval 10–105 °C. To ensure the preservation of Fe‐Met80 coordination, the experiments were carried out at pH 7.4. The complex behavior of CD and fluorescence spectra required principal component analysis (PCA) in which the first principal component (P1) of spectra was fitted to a three‐state or four‐state model, depending on the assay (Fig. S5). The fitting of P1 provided *T*_m_ values for the different thermal transitions that the mutants underwent along the temperature ramp (Table 2).

**Table 2:** Midpoint melting temperature (*T*_m_) values for oxidized WT and mutant cytochrome *c* as calculated within the 185–250 nm (UV) and 300–600 nm (Vis) ranges by CD and fluorescence spectroscopy.

	*T*_m_ (°C)		
	CD spectra		Fluorescence
	185–250 nm	300–600 nm	185–250 nm
WT	55.5 ± 0.8/87.1 ± 0.2	62.4 ± 1.1/87.4 ± 0.3	48.6 ± 2.4/58.2 ± 0.9/93.4 ± 1.6
K8A	52.5 ± 0.2/86.5 ± 0.2	28.5 ± 0.8/56.1 ± 0.6/84.1 ± 1.2	53.2 ± 0.4/88.4 ± 0.4
K8Q	53.2 ± 0.3/88.2 ± 0.4	29.1 ± 0.3/69.1 ± 3.7/87.8 ± 0.7	51.2 ± 0.3/90.1 ± 0.3
K53A	57.7 ± 4.6/82.1 ± 1.7	49.0 ± 0.8/83.5 ± 0.2	52.4 ± 0.6/88.8 ± 2.5
K53Q	55.7 ± 0.4/87.8 ± 0.3	53.6 ± 0.5/87.0 ± 0.6	57.6 ± 0.5/92.5 ± 0.3

The thermal unfolding profile from the representation of P1 and temperature in the far‐UV CD spectra of all mutants showed similar thermal behavior to the WT protein and consisted of two thermal transitions (Fig. S5). These thermal unfolding profiles have been explained using a previous folding model, based on the division of C*c* into five folding regions (Fig. 1) with different thermal stabilities [67]. According to the model, the most stable region included helix I and helix IV (foldon I), while the Ω‐loop between Thr19 and Phe36 residues and helix III comprised the second folding unit (foldon II). Denaturation of these two folding units may correspond to the second thermal transition observed in the far‐UV CD spectra, since foldons I and II are helix‐rich regions. The following region (foldon III) corresponds to two short stretches of residues from Phe36 to Lys39 and Gly56 to Gly60, while foldon IV includes the loop extending between helix III and helix IV and contains Met80 residue. The most unstable region (foldon V) extends from Thr40 to Lys55, between the two stretches of foldon III. The configuration of these three last regions is less structured and may thus be responsible for the first thermal transition. Since the Lys8 and Lys53 residues are located in helix I in foldon I and helix II in foldon V, respectively, preservation of the C*c* thermal profile in the far‐UV range indicates that the mutation of these residues does not alter foldon stability. The *T*_m_ values for WT and mutant C*c* species were quite similar (Table 2), with small differences consistent with the minor changes in secondary structure previously described in Figs 4 and S2.

However, the P1 plot of the near‐UV/Vis CD spectra showed that the Lys8 mutations, especially in the K8A mutant, considerably modified the thermal behavior of C*c*. While structural features of the heme moiety, such as heme plane bending, aromatic residue packing, and heme internal electric field [68, 69], determine the visible B‐band spectra, the thermal profile variations in the near‐UV/Vis CD region could correspond to conformational changes in the heme crevice. The appearance of a new thermal transition at ca. 28 °C indicates the influence of the Lys8 mutation on the heme moiety (Fig. S5, Table 2). This residue is located in helix I in foldon I, which is where the Cys14 and Cys17 residues are located. Cys14 and Cys17 are covalently linked to the heme group, and so perturbations of the foldon I residues could affect the redox cofactor. This possible alteration of the redox cofactor was not predicted by theoretical MD calculations (Fig. S4), which yielded a value at 25 °C lower than that of the new thermal transition. On the other hand, there is no substantial evidence of changes in the thermal profile after Lys53 mutation. The transitions for WT and Lys53 mutants in the near‐UV/Vis CD region are consistent with the equivalent ones in the far‐UV CD region. The *T*_m_ values of Lys53 variants differed slightly from those of the WT form (Table 2), a fact that can be attributed to small heme moiety variations resulting from the changes in C*c* conformation, mentioned below.

Trp59 fluorescence of C*c*, which is quenched by the heme porphyrin ring in the native state, was recorded [70]. Although the fluorescence profiles of Lys8 mutants are close to that of the WT form, those of Lys53 mutants showed differences at temperatures between 60 and 80 °C (Fig. S5). Since Lys53 and Trp59 are located in contiguous regions (foldons V and III, respectively), mutation at Lys53 could destabilize foldon V and, in turn, foldon III, causing the change in Trp59 fluorescence. The final decrease in fluorescence intensity of all C*c* species probably corresponds to the exposure of the Trp59 indole ring to the solvent as the protein unfolds [71]. The fitting of P1 fluorescence gave three *T*_m_ values for WT and only two for C*c* mutants (Table 2). A detailed analysis of the fluorescence P1 curves of mutants revealed the coupling of two close low‐temperature transitions that cannot be distinguished with the model used.

Taken together, these findings suggest that the thermal stability of C*c* is affected differently depending on the mutated position, since Lys8 and Lys53 are located on opposite faces of the C*c* surface.

### Mimicking acetylation induces local structural changes in cytochrome c

**Fig. 5:**
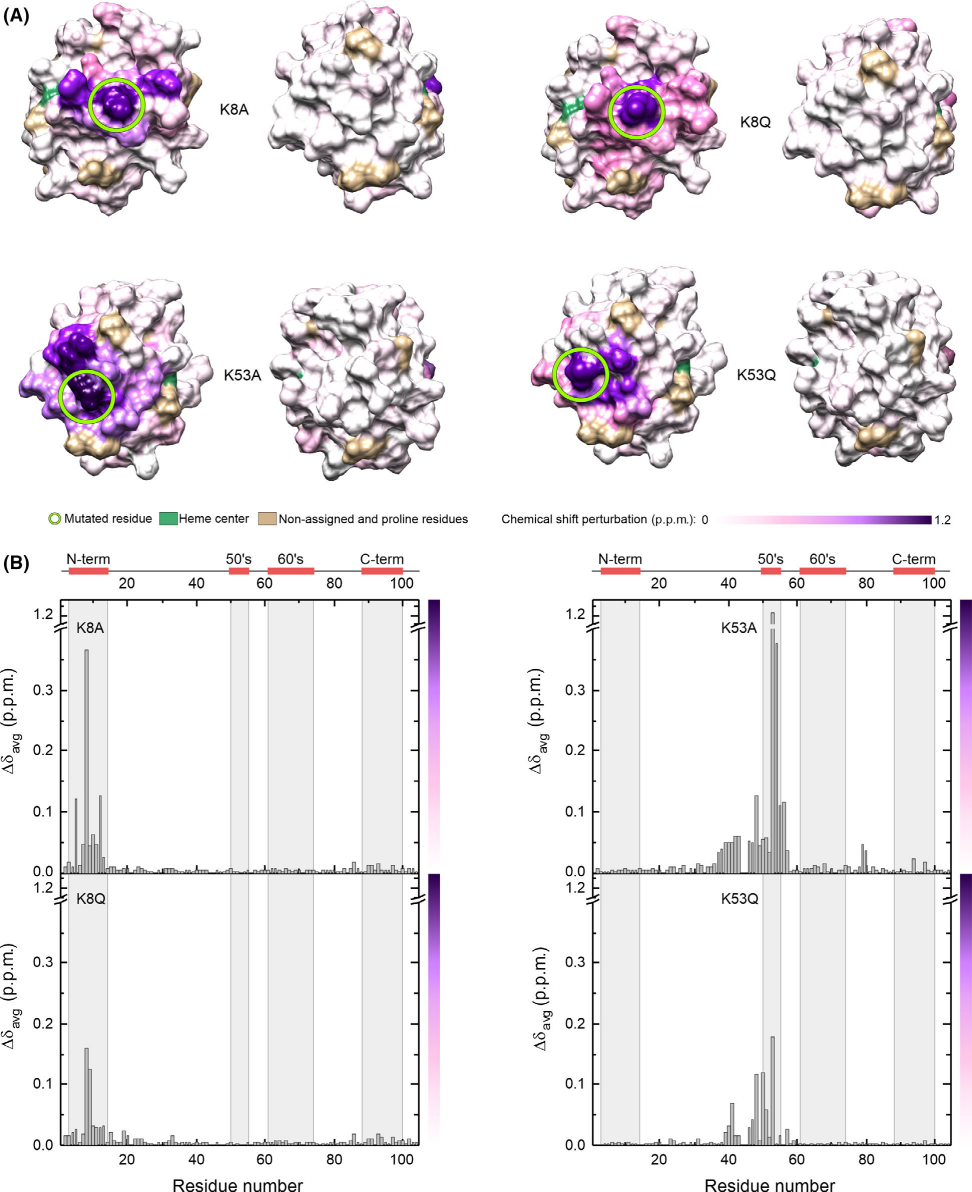
NMR chemical shift perturbations of reduced cytochrome *c* mutants. (A) Each surface map shows residues colored according to CSPs calculated previously from 2D ^1^H‐^15^N HSQC spectra of reduced K8A, K8Q, K53A, and K53Q C*c* with respect to the 2D ^1^H‐^15^N HSQC spectra of reduced WT C*c*. (B) Plot of NMR CSPs of reduced C*c* mutants. CSP values as a function of residue number calculated from 2D ^1^H‐^15^N HSQC spectra of reduced K8A, K8Q, K53A, and K53Q C*c* with respect to the 2D ^1^H‐^15^N HSQC spectrum of reduced WT C*c*. The color bar represents the color scale used in panel A.

To understand the effect of the point mutations on the spatial structure of C*c*, the reduced forms of K8A, K8Q, K53A, and K53Q C*c* mutants were characterized by recording the 1D ^1^H, 2D ^1^H–^15^N HSQC, 3D ^15^N‐^1^H TOCSY‐HSQC, and 3D ^15^N‐^1^H NOESY‐HSQC NMR spectra to monitor the backbone amide signals and compare them with those from the WT protein. The 1D ^1^H NMR spectra of all mutants showed a resonance at ca. −3.5 ppm, which corresponds to the ^1^H signal of Met80‐ε‐CH_3_ group and corroborates the preservation of native heme axial coordination, in accordance with previous data. The backbone amide resonances of the C*c* mutants identified in 2D ^1^H‐^15^N HSQC spectra were confirmed by recording 3D ^15^N‐^1^H TOCSY‐HSQC and 3D ^15^N‐^1^H NOESY‐HSQC spectra for each of the mutants. The difference in CSPs calculated from the 2D ^1^H‐^15^N HSQC of each reduced mutant with respect to that of the reduced WT is illustrated in Fig. 5. Generally speaking, Lys‐to‐Ala mutation results in substantial CSPs compared with Lys‐to‐Gln one; not only in terms of the magnitude of the CSP but also the number of affected residues. These differences can be followed in Fig. S6–S9. A plausible explanation is that the Lys‐to‐Ala substitution induces significant changes in the chemical environment of those residues close to the new alanine residue, which differs from lysine in a loss of charge and side‐chain length [72, 73]. On the other hand, the chemical environment is less affected by the Lys‐to‐Gln substitution, albeit with new resonances corresponding to the appearance of side‐chain amide groups of Gln in the NMR spectra (Figs S7 and S9.

A surface map comparison of NMR CSPs of acetyl‐mimetic C*c* mutants revealed that the K8Q spectrum showed a smaller number of affected residues with respect to the WT protein (Fig. 5). In K8Q, only Lys5, Ile9, Phe10, Ile11, and Met12 (located in the same helix I as Gln8) experienced significant CSPs (Fig. S7). However, the superposition of the K53Q and WT HSQC spectra (Fig. S9) revealed that the loss of charge at position 53 caused by the mutation drastically alters the chemical environment of nearby residues in the sequence and close amino acidic neighbors in the space. Some of them (Thr40, Gly41, Gln42, Ser47, and Tyr48) are located in the nested Ω‐loop, with the side chain of Gln53 pointing toward it. Like K8Q, other residues located in the same helix II as Gln53 (Ala50, Ala51, and Asn54, including its amide side chain) undergo large CSPs (Fig. S9). Consistent with the thermal stability assays, the spatial location of the mutated residues in the protein structure could explain the functional changes shown by the acetyl‐mimetic mutants.

### Binding between the acetyl‐mimetic cytochrome c species and its redox partner cytochrome c_1_ is subtly dependent on the spatial location of the mutated residues

The interaction of C*c* with its metabolic redox and non‐redox partners takes place mainly through the Gln16, Ala50, Ala51, Lys72, Gly77, Ile81, and Lys86 residues, which surround the heme crevice and constitute the binding surface. It was previously reported that lysines 8, 13, 27, 72, 86, and 87 are all in contact with C*c*_1_, whereas lysines 5, 7, 8, 22, 25, 53, 72, 73, 86, and 88 contact the SET/TAF‐Iβ histone chaperone. [16, 22, 34, 37]. Considering that Lys8 and Lys53 are involved in C*c* binding to its partners, one would expect this interaction to be modulated by mutation of those residues.

Our previous NMR data on the plant C*c*_1_‐human C*c* complex indicated that lysine at position 8 is substantially perturbed by binding to C*c*_1_, suggesting that it is involved in interface formation [16]. The question arises whether acetyl‐mimetic C*c* could alter C*c* binding to C*c*_1_. In this context, BLI was used to estimate the binding affinity values for the plant C*c*_1_‐human C*c* complex. The N‐terminal soluble domains of human and plant C*c*_1_ share a 62% of sequence identity and similar charge distributions. Previous NMR and Isothermal titration calorimetry (ITC) analyses indicated that *Arabidopsis thaliana* C*c*_1_ shows almost identical affinity toward human and *Arabidopsis thaliana* C*c* [15, 16]. The maximum wavelength shifts at saturation were plotted against protein concentration and fitted to a non‐linear equation to calculate the *K*_D_ (Fig. S10). The *K*_D_ values obtained for the WT protein and the Lys53 acetyl‐mimetic variant were similar (Table 3), although the binding profile for K53Q C*c* suggests the existence of two binding events. However, the *K*_D_ value for the C*c*_1_‐K8Q C*c* complex was slightly lower than for the WT complex. This could be explained by the fact that Lys8 is directly involved in the interaction with C*c*_1_ [16, 74].

**Table 3:** Dissociation constant (*K*_D_) values for the interaction of reduced cytochrome *c*_1_ with reduced WT, K8Q, and K53Q cytochrome *c*.

	*K*_D_ (µm)
WT	0.137 ± 0.032
K8Q	0.099 ± 0.004
K53Q	0.116 ± 0.014

### Thermodynamics and kinetics of interfacial electron transfer in cytochrome c mutants

It has been shown that PTMs of C*c* are able to regulate its electron donor function [14, 32, 39, 54, 75, 76, 77, 78, 79, 80]. Since the patch of positively charged lysine residues of C*c* contributes to electrostatic interactions with redox partners in mammals, plants, and yeasts [15, 16, 34, 35, 37, 74, 81, 82, 83, 84], the redox functionality of C*c* may be influenced by lysine mutation.

Consequently, the redox functionality of acetyl mimetics of C*c* in the adsorbed state was also assessed. C*c* actually performs its many biological functions upon partner binding, so protein film voltammetry was used as a suitable technique to get further information on the redox properties of bound C*c*. A common strategy consists in the immobilization of the redox protein onto a metal electrode modified with SAM of organic molecules to reproduce the essential features of protein/protein interfaces. For this, WT, K8Q, and K53Q C*c* species were adsorbed onto polycrystalline gold electrodes modified with mixed SAMs made up of 1 : 2.5 mixtures of MOA and MOOL to mimic the biological interface of C*c* in its primary redox function. The thermodynamics and kinetics of interfacial electron transfer were determined by performing variable temperature cyclic voltammetry experiments. The estimated characteristic interfacial electron transfer parameters are shown in Table 4.

**Table 4:** Midpoint redox potential, thermodynamic and kinetic parameters as calculated from electron transfer of WT, K8Q, and K53Q cytochrome *c* immobilized on a chemically modified polycrystalline gold electrode. $E_{\frac{1}{2}}$, Midpoint redox potential value, $\Delta S_{\mathrm{rc}}^{0}$, Reduction entropy, $\Delta H_{\mathrm{rc}}^{0}$, Reduction enthalpy, *k*_s_, Electron transfer rate constant, A, Pre‐exponential factors, $\Delta H_{\mathrm{ET}}^{\#}$, Activation enthalpy.

	$E_{\frac{1}{2}}$ ^a^ (mV vs. NHE)	$\Delta S_{\mathrm{rc}}^{0}$ (J K^−1^·mol^−1^)	$\Delta H_{\mathrm{rc}}^{0}$ (kJ·mol^−1^)	*k*_s_ (s^−1^)	10^‐5^·A (s^−1^)	$\Delta H_{\mathrm{ET}}^{\#}$ (kJ·mol^−1^)
WT	184 ± 5	–142 ± 8	–60 ± 6	1300 ± 50	3.1 ± 0.9	13.6 ± 0.5
K8Q	178 ± 5	–130 ± 7	–56 ± 4	1100 ± 50	12.1 ± 1.8	17.2 ± 0.8
K53Q	172 ± 5	–108 ± 8	–49 ± 5	1600 ± 50	9.7 ± 1.3	15.8 ± 0.7

^a^ Measured at 25 °C.

The WT, K8Q, and K53Q C*c* species showed the typical voltammetric response of an isolated population of surface‐immobilized species (Fig. S11A). The standard potential of the WT C*c* species at 25 °C was determined from the average of the cathodic and anodic peak potentials of the experimental voltammograms at low potential scan rate (0.1 V·s^−1^). The standard potential associated with the Fe^3+^ / Fe^2+^ redox conversion of the heme group was ˜ 185 mV vs. NHE, which was significantly more negative than the one in solution (˜ 265 mV) [85], revealing a further relative stabilization of the ferric form of the native heme conformation upon electrostatic immobilization. The midpoint redox potential values (*E*_1/2_) at 25 °C for the three C*c* species were not significantly different (Table 4), thus suggesting that the acetyl‐mimetic mutations do not substantially modify the heme moiety.

The variation of the standard redox potential with temperature (Fig. S11B) provided the reduction entropy ($\Delta S_{\mathrm{rc}}^{0}$) and enthalpy ($\Delta H_{\mathrm{rc}}^{0}$) values for the redox conversion of immobilized proteins (Table 4). More specifically, the $\Delta S_{\mathrm{rc}}^{0}$ and $\Delta H_{\mathrm{rc}}^{0}$ values were estimated from the slopes of *E*_1/2_ vs. temperature (*T*) and *E*_1/2_/T vs. 1/*T* plots, respectively. The redox thermodynamics of the adsorbed C*c* species turned out to be characterized by highly negative $\Delta S_{\mathrm{rc}}^{0}$ and $\Delta H_{\mathrm{rc}}^{0}$ values, revealing large differences in protein solvation and/or structure after its change in oxidation state. It can also be observed that the $\Delta S_{\mathrm{rc}}^{0}$ and $\Delta H_{\mathrm{rc}}^{0}$ values were relatively insensitive to the mutation. For electron transfer proteins with positively charged redox centers, less negative reduction entropies are generally associated with the greater role of solvent reorganization accompanying the electron transfer step. The less negative $\Delta H_{\mathrm{rc}}^{0}$ values of the mutants point either to an increase in water accessibility to the heme, which was not predicted for the proteins in solution (Fig. S4), or to a perturbation of the Fe‐ligand bond favoring the ferric over the ferrous heme. Notably, the perfect enthalpy‐entropy compensation observed in the three proteins also points to the substantial contribution of solvent exchange between protein and solution to the energetics of protein reduction.

The interfacial electron transfer kinetics of adsorbed C*c* species was also explored by determining their standard heterogeneous electron transfer rate constants ($k_{\mathrm{ET}}^{0}$). To obtain the activation parameter of the electron transfer process, we investigated the temperature dependence of $k_{\mathrm{ET}}^{0}$ for immobilized C*c* species (Fig. S11C). Pre‐exponential factors (A) and activation enthalpies ($\Delta H_{\mathrm{ET}}^{\#}$) obtained from the intercept and slopes of the linear Arrhenius plots are shown in Table 4. The electron exchange rates between electrode and the different C*c* species were essentially the same. Interestingly, the glutamine mutation drives a markedly higher activation enthalpy, whose effect is partially offset by an increase in the pre‐exponential factor. The higher $\Delta H_{\mathrm{ET}}^{\#}$ values obtained for the mutants are consistent with greater solvent participation in the activation process [86], whereas their higher A values could come from the increased dynamics of the immobilized K8Q and K53Q mutants.

**Fig. 6:**
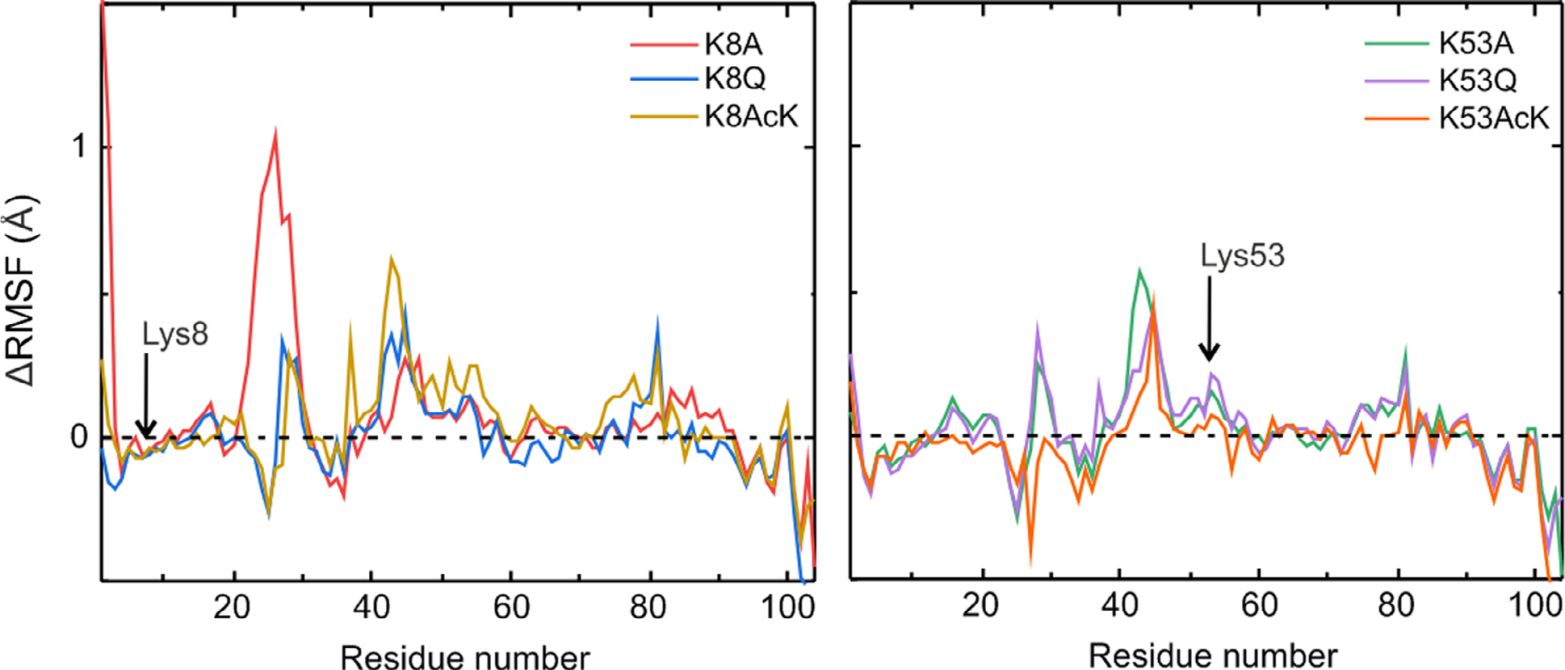
Simulation of molecular fluctuations in cytochrome *c* species. Comparison of the ∆RMSF of reduced K8A, K8Q, K8AcK, K53A, K53Q, and K53AcK mutants with respect to reduced WT C*c* over the last 50 ns of their respective MD trajectories.

Analyses of trajectories from MD simulations revealed that both the overall folding and heme environment are very well preserved in the two C*c* mutants. RMSD and radius of gyration (RG) values also displayed a similar behavior (Table 5), which indicates the absence of mutation‐induced protein unfolding or significant structural changes. The close similarity of the heme group solvent‐accessible surface and the Met80‐Fe distance in WT and mutant C*c* species indeed confirmed that the heme environment is practically unaltered (Fig. S4), in agreement with visible CD spectra and *E*_1/2_ values. Interestingly, the different values for atomic root mean square fluctuation (∆RSMF) obtained with the mutants as compared with WT C*c* revealed an increase in protein dynamics of residues at the interaction surface of C*c* with its metabolic partners (Fig. 6). In particular, the RMSF values of the 25–35, 40–60, and 72–85 stretches in the two acetyl mimetics are higher than those of WT C*c*, explaining the increase in the pre‐exponential factor of immobilized acetyl‐mimetic mutants.

**Table 5:** Statistics of molecular dynamics (MD) simulation trajectories with WT and mutant C*c* species. Average values were calculated for the plateau of the MD simulation (last 50 ns). For all simulations, RMSD drift was smaller than 0.003 Å/ns.

	Average RMSD (Å)	Average RG (Å)	Average SAS (Å^2^)	Met80‐Fe distance (Å)
C*c* WT	0.79 ± 0.14	12.93 ± 0.05	49.68 ± 16.26	2.46 ± 0.03
C*c* K8A	1.00 ± 0.21	12.94 ± 0.05	57.19 ± 14.89	2.46 ± 0.03
C*c* K8Q	0.87 ± 0.16	12.98 ± 0.05	51.78 ± 18.50	2.46 ± 0.03
C*c* K8AcK	0.92 ± 0.17	13.00 ± 0.05	52.66 ± 18.24	2.45 ± 0.03
C*c* K53A	0.90 ± 0.17	13.00 ± 0.05	60.15 ± 12.71	2.45 ± 0.03
C*c* K53Q	0.97 ± 0.19	12.99 ± 0.04	48.20 ± 17.64	2.45 ± 0.03
C*c* K53AcK	0.91 ± 0.16	12.99 ± 0.05	40.27 ± 13.45	2.45 ± 0.03

**Fig. 7:**
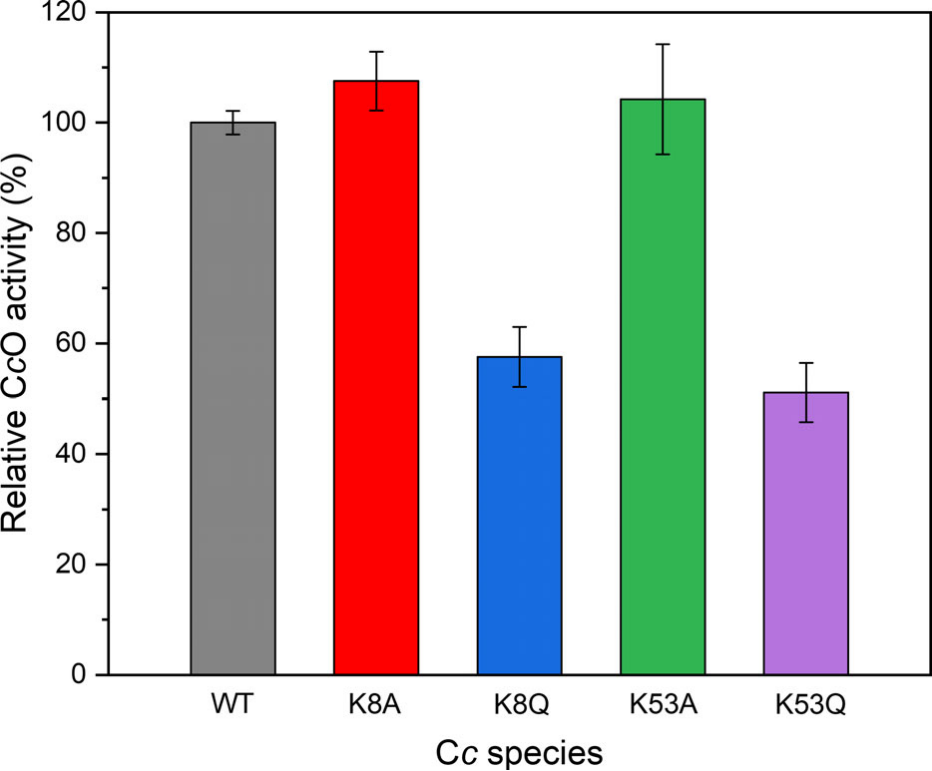
Cytochrome *c* oxidase activity with WT and mutant cytochrome *c* as electron donors. C*c*O activity was determined in mitochondria isolated from MEF cells upon addition of exogenous WT, K8Q, K8A, K53Q, and K53A C*c*. Experimental data were normalized with respect to that obtained with WT C*c*. Each value represents the average of three individual experiments ± SD. See the Methods section for details.

The impact of Lys8 and Ly53 mutations on the electron donor capacity of C*c* in the mitochondrial context (where the hemeprotein acts as mediator between CIII and CIV) was also explored. Mitochondrial C*c*O activity assays revealed that electron flow was significantly lower with the two acetyl mimetics than with the WT C*c* species (Fig. 7). These data are consistent with the *in vitro* electron transfer rate of K53Q to CIV recorded by Bazylianska and colleagues [32] and the involvement of Lys8 at the interface of the C*c*‐CIV complex reported previously [36, 74, 84, 87]. It has actually been published that there are two binding sites for C*c*—the so‐called *proximal* and *distal* sites—on the surface of CIV, where the surface residues exposed to the binding interface differ [15]. A detailed analysis of this binding interface showed the involvement of Lys8 and Lys53 residues at the *proximal* and *distal* C*c*‐binding sites, respectively, in the C*c*‐CIV complex (Fig. S12). This finding suggests that mutation of Lys8 could modulate electron transfer in the C*c*‐CIV complex, whereas mutation of Lys53 could slow down the turnover of C*c* molecules. Although electron carriers display a certain degree of ‘promiscuity’ due to the fact that their surfaces are often recognized by multiple partners, their redox centers are usually close to a hydrophobic patch that optimizes electron transfer between redox partners, as is the case in a high number of metabolic processes and reactions [88, 89, 90, 91, 92, 93, 94, 95]. Hence, the observed alteration of electron transfer to CIV when Lys8 or Lys53 are modified can be explained by a direct or indirect modification of the recognition mechanism between the two partners [36].

## Conclusion

Structural and functional analysis of site‐directed acetyl‐mimetic mutants is a helpful approach to acetylated C*c* characterization. Our CD and Vis spectroscopy results indicated that acetyl‐mimetic mutants at residues Lys8 and Lys53 keep the overall physicochemical features of C*c*. However, the thermal stability of C*c* species is slightly altered, with changes dependent on the spatial location of mutated residue. The observed differences may be related to the backbone resonance changes detected by NMR, since the magnitude and number of affected residues also depend on the position of the mutated amino acid.

Interestingly, mimetic acetylation enhances C*c* dynamics and solvent involvement in the redox process in the immobilized state, as observed by cyclic voltammetry. With respect to redox functionality, both mimetic C*c* species exhibit a significant decrease in C*c*O activity in a mitochondrial context, suggesting that these residues are involved in the interaction with CIV and, consequently, that alteration of protein functionality by lysine acetylation is likely. On the other hand, when binding to the C*c*_1_ partner, only the Lys8 mutants show an increased binding affinity for the C*c*_1_‐C*c* complex; this can be attributed to the fact that Lys8 is in direct contact with C*c*_1_.

These results lay the groundwork for more detailed studies on the effect of acetylation on the multiple extra‐mitochondrial functions of C*c* and its implication for the development of certain diseases.

## Conflict of interest

All authors declare no conflict of interest.

## Author contributions

All authors contributed to conception and experimental design, discussed findings and wrote the manuscript. IM, GPM, AGC and JLOS performed experimental research work.

## Supporting information

Fig. S1. Expression and purification of WT and mutant cytochrome *c* species. (A) *Left* panel: SDS‐PAGE of the ^15^N‐labelled WT, K8A, K8Q, K53A and K53Q C*c* samples used for NMR experiments (5 μg of protein loaded in each lane). The bands within the red rectangle (below 15 kDa) correspond to the different C*c* species. M: Molecular mass markers. *Right* panel: Western blot of purified WT, K8A, K8Q, K53A and K53Q C*c* showing the detection of the C*c* band in each lane. (B) Tryptic digestion of proteins extracted from the bands of an SDS‐PAGE similar to that in (A) but loaded with ^14^N‐labelled C*c* samples. The calculated masses of AIFIMK, QIFIMK, KTGQAPGYSYTAANANK and KTGQAPGYSYTAANQNK fragments are 722, 779, 1741 and 1799 Da, respectively.
Fig. S2. Secondary structure analysis of WT and mutant cytochrome *c* species. Percentage of secondary structure for oxidized WT, K8A, K8Q, K53A and K53Q C*c* is calculated from far‐UV CD data with the CDPro software package (SP43, SMP50 and CLSTR reference sets) [1]. The results are expressed as the mean ± SD.
Fig. S3. Alkaline transition of WT and mutant cytochrome *c* species. Electronic absorption spectra were recorded at different pH values (*left* panels), and titration curves were determined by following the absorbance changes at 695 nm (*right* panels) of the oxidized species. Process reversibility was checked by recording the last spectrum at the initial pH value. The full data set was fitted to the Henderson–Hasselbalch equation to calculate the p*K*_a_ values (red lines; see details in Methods section).
Fig. S4. Molecular dynamic simulations of WT and mutant cytochrome *c* species. Average of the Met80Fe distance (*upper*) and solvent accessibility of the heme group (*lower*) within the last 50 ns of their respective MD trajectories are shown for WT, K8A, K8Q, K8AcK, K53A, K53Q and K53AcK C*c* species. The results are expressed as the mean ± SD.
Fig. S5. Thermal stability of WT and mutant cytochrome *c* species. The first principal component (P1) of CD and fluorescence spectra of oxidized WT, K8A, K8Q, K53A and K53Q C*c* recorded at varying temperature is plotted.
Fig. S6. Superimposition of 2D ^1^H–^15^N HSQC NMR spectra of WT (black) and K8A (red) cytochrome *c*. *Insets*: Zooms of the signals framed in yellow boxes. The green arrows stand for the CSPs of mutant C*c* as compared with the WT species. The yellow circle denotes the mutated residue.
Fig. S7. Superimposition of 2D ^1^H–^15^N HSQC NMR spectra of WT (black) and K8Q (blue) cytochrome. *Insets*: Zooms of the signals framed in yellow boxes. The red arrows stand for the CSPs of mutant C*c* as compared with the WT species. The red dotted line shows the ^15^N chemical shift at which the ^1^H of the Q8 ε‐amine group appears. The yellow circle denotes the mutated residue.
Fig. S8. Superimposition of 2D ^1^H–^15^N HSQC NMR spectra of WT (black) and K53A (green) cytochrome *c*. *Insets*: Zooms of the signals framed in yellow boxes. The red arrows stands for the CSPs of mutant C*c* as compared with the WT species. The yellow circle surrounds the mutated residue.
Fig. S9. Superimposition of 2D ^1^H–^15^N HSQC NMR spectra of WT (black) and K53Q (purple) cytochrome *c*. *Insets*: Zooms of the signals framed in yellow boxes. The green arrows stand for the CSPs of mutant C*c* as compared with the WT species. The green dotted line shows the ^15^N chemical shift at which the ^1^H of the Q53 ε‐amine group appears. The yellow circle surrounds the mutated residue.
Fig. S10. Interaction of WT and mutant cytochrome *c* with cytochrome *c*_1_ as determined by BLI. Wavelength shift over time (*left* panels) and calculated dissociation equilibrium constant (*K*_D_; *right* panels) for the interaction between reduced C*c*_1_ and reduced WT, K8Q or K53Q C*c* were determined at increasing C*c* concentration. The dotted gray line in the left panels indicates the time at which the wavelength shifts were taken for calculation of the *K*_D_ values in the right panels. The data set was fitted to a non‐linear equation to obtain the *K*_D_ values (red lines; see details in Methods section). The results are expressed as the mean ± SD.
Fig. S11. Electrochemical characterization of WT and mutant cytochrome *c*. (A) Cyclic voltammograms of reduced C*c* species immobilized onto a chemically modified polycrystalline gold electrode (scan rate: 0.1 V s^‐1^). (B) Midpoint redox potential and (C) electron transfer rate constant for immobilized C*c* as determined at varying temperature under the same experimental conditions as in panel A. The results are expressed as the mean ± SD.
Fig. S12. Binding sites for cytochrome *c* on complex IV at the respiratory supercomplex. The representative structure of WT C*c* at the *distal* site of complex IV is in orange, where Lys53 (green) points towards the complex IV surface (light gray), and that at the *proximal* site is in red, where Lys8 (blue) points towards the complex IV surface.
Table S1. Primers used in the design of cytochrome *c* mutants.
Table S2. BLI protocol for cytochrome *c*_1_ – cytochrome *c* interaction.

## Data Availability

The data that support the findings of this study are available from the corresponding author upon reasonable request.
