# Supplementary material for: Structural and functional insights into lysine acetylation of cytochrome c using mimetic point mutants
Source: FEBS Open Bio. 2021 Nov 9;11(12):3304–23. doi: 10.1002/2211-5463.13284 (PMC8634867; doi:10.1002/2211-5463.13284)
Supplement: Supplementary file 1 — Fig. S1. Expression and purification of WT and mutant cytochrome c species. (A) Left panel: SDS‐PAGE of the 15N‐labelled WT, K8A, K8Q, K53A and K53Q Cc samples used for NMR experiments (5 μg of protein loaded in each lane). The bands within the red rectangle (below 15 kDa) correspond to the different Cc species. M: Molecular mass markers. Right panel: Western blot of purified WT, K8A, K8Q, K53A and K53Q Cc showing the detection of the Cc band in each lane. (B) Tryptic digestion of proteins extracted from the bands of an SDS‐PAGE similar to that in (A) but loaded with 14N‐labelled Cc samples. The calculated masses of AIFIMK, QIFIMK, KTGQAPGYSYTAANANK and KTGQAPGYSYTAANQNK fragments are 722, 779, 1741 and 1799 Da, respectively. Fig. S2. Secondary structure analysis of WT and mutant cytochrome c species. Percentage of secondary structure for oxidized WT, K8A, K8Q, K53A and K53Q Cc is calculated from far‐UV CD data with the CDPro software package (SP43, SMP50 and CLSTR reference sets) [1]. The results are expressed as the mean ± SD. Fig. S3. Alkaline transition of WT and mutant cytochrome c species. Electronic absorption spectra were recorded at different pH values (left panels), and titration curves were determined by following the absorbance changes at 695 nm (right panels) of the oxidized species. Process reversibility was checked by recording the last spectrum at the initial pH value. The full data set was fitted to the Henderson–Hasselbalch equation to calculate the pK a values (red lines; see details in Methods section). Fig. S4. Molecular dynamic simulations of WT and mutant cytochrome c species. Average of the Met80Fe distance (upper) and solvent accessibility of the heme group (lower) within the last 50 ns of their respective MD trajectories are shown for WT, K8A, K8Q, K8AcK, K53A, K53Q and K53AcK Cc species. The results are expressed as the mean ± SD. Fig. S5. Thermal stability of WT and mutant cytochrome c species. The first principal component (P1) of CD and [file FEB4-11-3304-s001.docx]

**Appendix A. Supporting information**

**Structural and functional insights into lysine acetylation of cytochrome *c* using mimetic point mutants**

Inmaculada Márquez^1^, Gonzalo Pérez-Mejías^1$^. Alejandra Guerra-Castellano^1$^, José Luis Olloqui‑Sariego^2^, Juan José Calvente^2^, Rafael Andreu^2^, Miguel A. De la Rosa^1^ and Irene Díaz-Moreno^1,^*

^1^Instituto de Investigaciones Químicas, Universidad de Sevilla – CSIC, Avda. Américo Vespucio 49, 41092 Sevilla, Spain

^2^Departament of Physical Chemistry, University of Seville, Sevilla, Spain

^$^These authors contributed equally to the manuscript.

***Correspondence**: I. Díaz-Moreno, Institute for Chemical Research (IIQ), Scientiﬁc Research Centre Isla de la Cartuja (cicCartuja), University of Seville – CSIC, Avda. Américo Vespucio 49, Sevilla 41092, Spain

E-mail: idiazmoreno@us.es


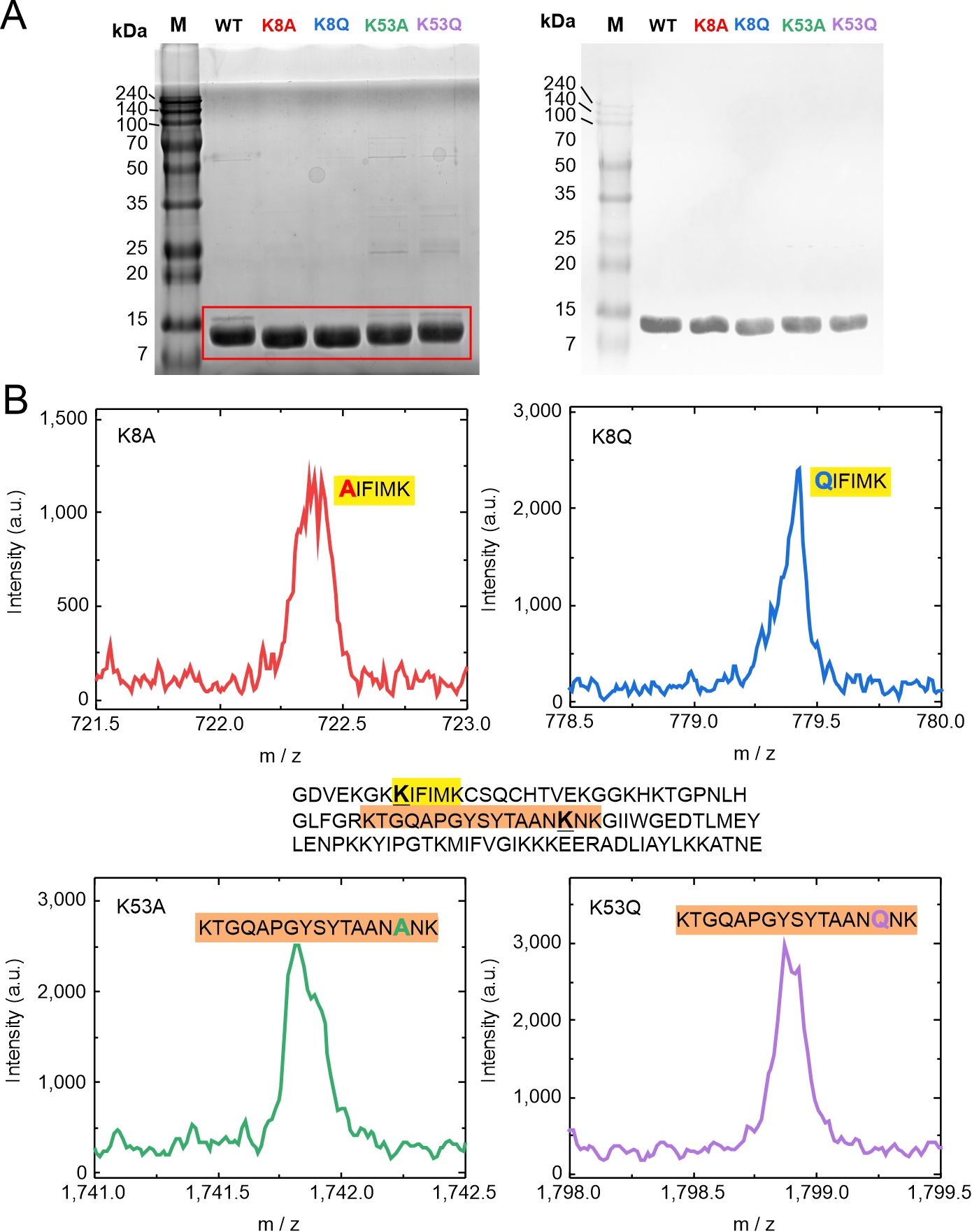


Figure S1. Expression and purification of WT and mutant cytochrome *c* species. (A) *Left* panel: SDS-PAGE of the ^15^N-labelled WT, K8A, K8Q, K53A and K53Q C*c* samples used for NMR experiments (5 μg of protein loaded in each lane). The bands within the red rectangle (below 15 kDa) correspond to the different C*c* species. M: Molecular mass markers. *Right* panel: Western blot of purified WT, K8A, K8Q, K53A and K53Q C*c* showing the detection of the C*c* band in each lane. (B) Tryptic digestion of proteins extracted from the bands of an SDS-PAGE similar to that in (A) but loaded with ^14^N-labelled C*c* samples. The calculated masses of AIFIMK, QIFIMK, KTGQAPGYSYTAANANK and KTGQAPGYSYTAANQNK fragments are 722, 779, 1,741 and 1,799 Da, respectively.


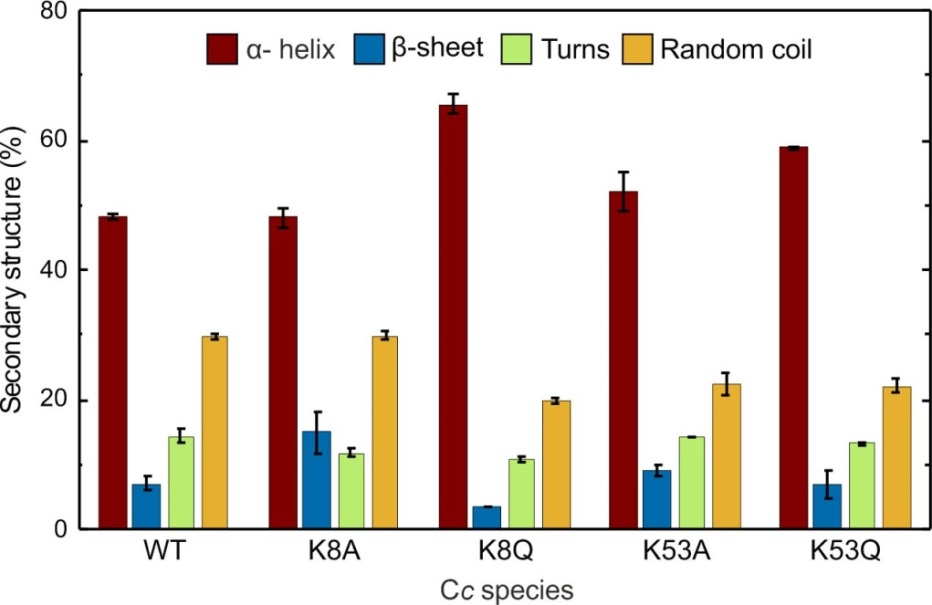


Figure S2. Secondary structure analysis of WT and mutant cytochrome *c* species. Percentage of secondary structure for oxidized WT, K8A, K8Q, K53A and K53Q C*c* is calculated from far-UV CD data with the CDPro software package (SP43, SMP50 and CLSTR reference sets) [1]. The results are expressed as the mean ± SD.


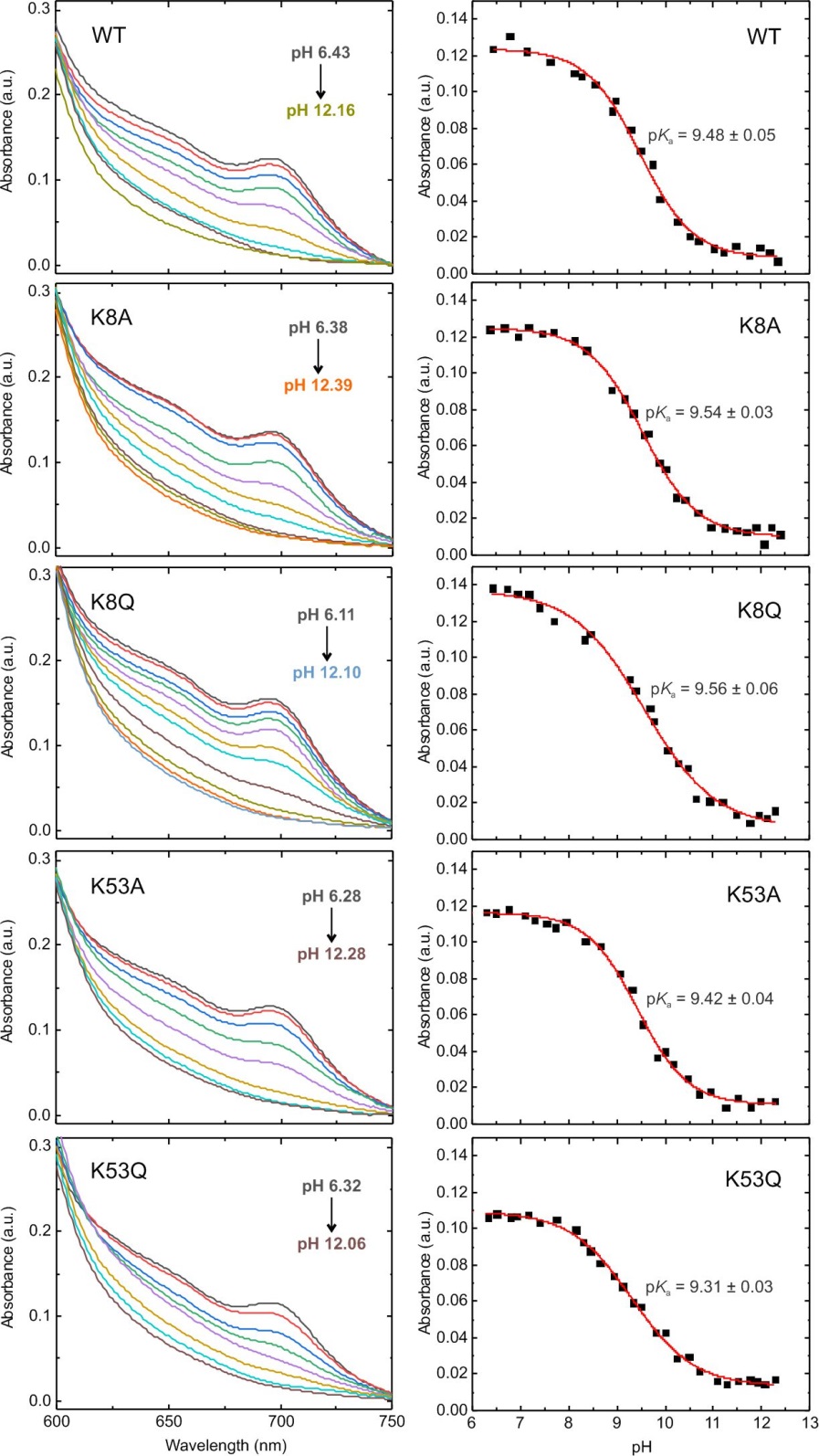


Figure S3. Alkaline transition of WT and mutant cytochrome *c* species. Electronic absorption spectra were recorded at different pH values (*left* panels), and titration curves were determined by following the absorbance changes at 695 nm (*right* panels) of the oxidized species. Process reversibility was checked by recording the last spectrum at the initial pH value. The full data set was fitted to the Henderson–Hasselbalch equation to calculate the p*K*_a_ values (red lines) (see details in Methods section).


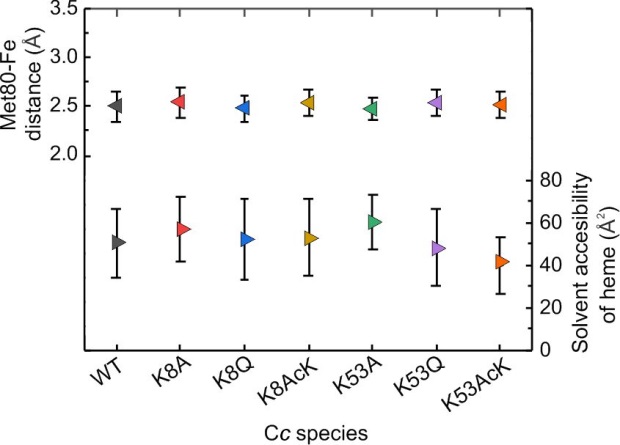


Figure S4. Molecular dynamic simulations of WT and mutant cytochrome *c* species. Average of the Met80‑Fe distance (*upper*) and solvent accessibility of the heme group (*lower*) within the last 50 ns of their respective MD trajectories are shown for WT, K8A, K8Q, K8AcK, K53A, K53Q and K53AcK C*c* species. The results are expressed as the mean ± SD.


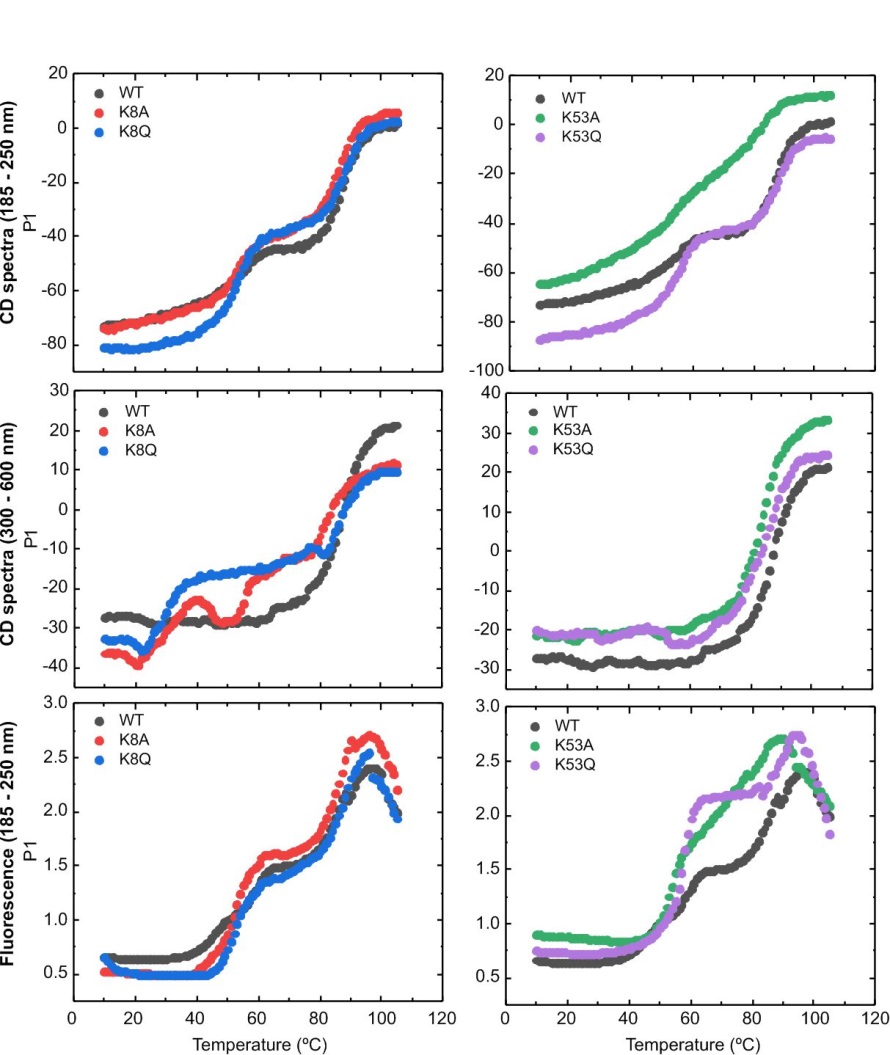


Figure S5. Thermal stability of WT and mutant cytochrome *c* species. The first principal component (P1) of CD and fluorescence spectra of oxidized WT, K8A, K8Q, K53A and K53Q C*c* recorded at varying temperature is plotted.


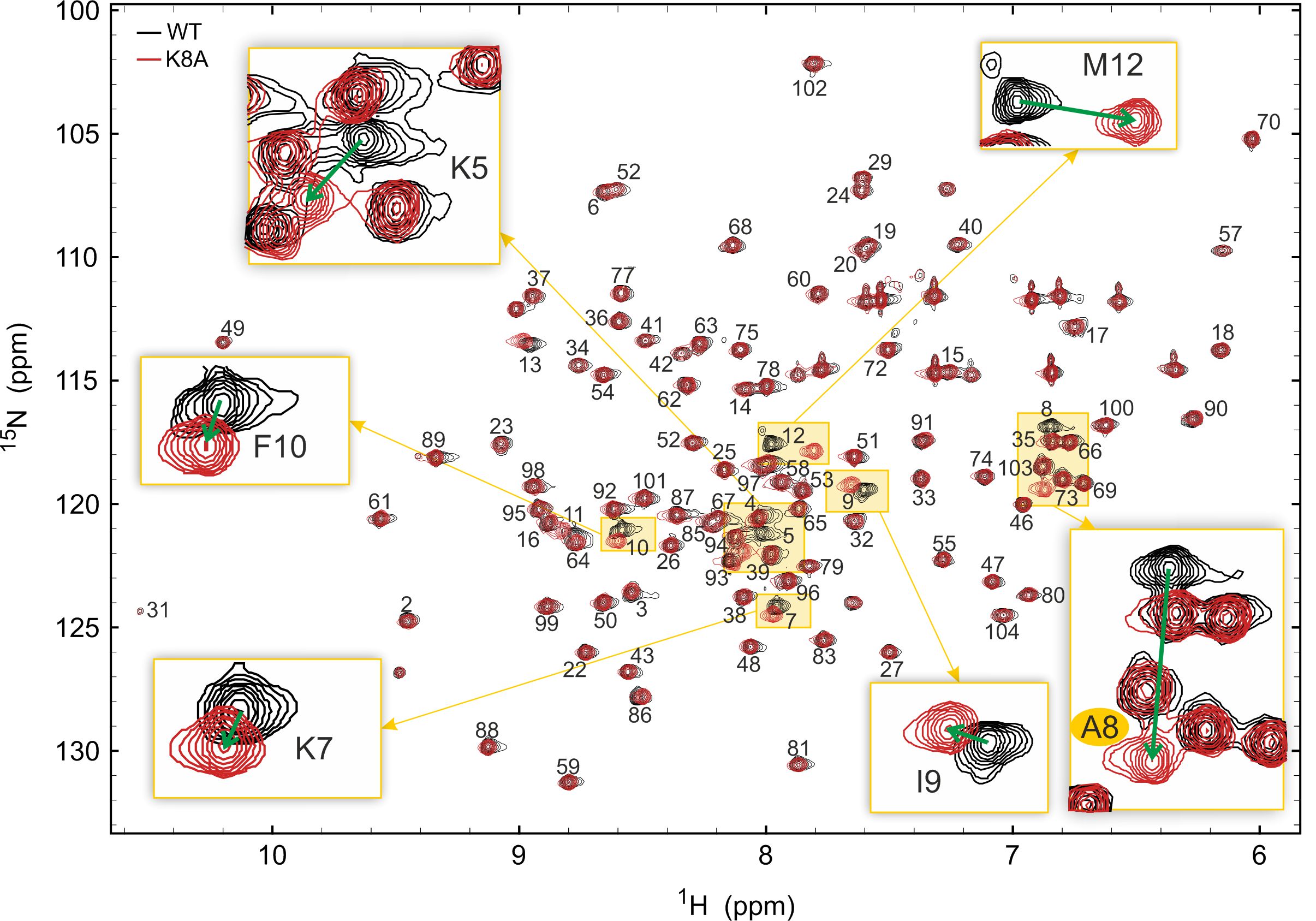


Figure S6. Superimposition of 2D ^1^H–^15^N HSQC NMR spectra of WT (black) and K8A (red) cytochrome *c*. *Insets*: Zooms of the signals framed in yellow boxes. The green arrows stand for the CSPs of mutant C*c* as compared with the WT species. The yellow circle denotes the mutated residue.


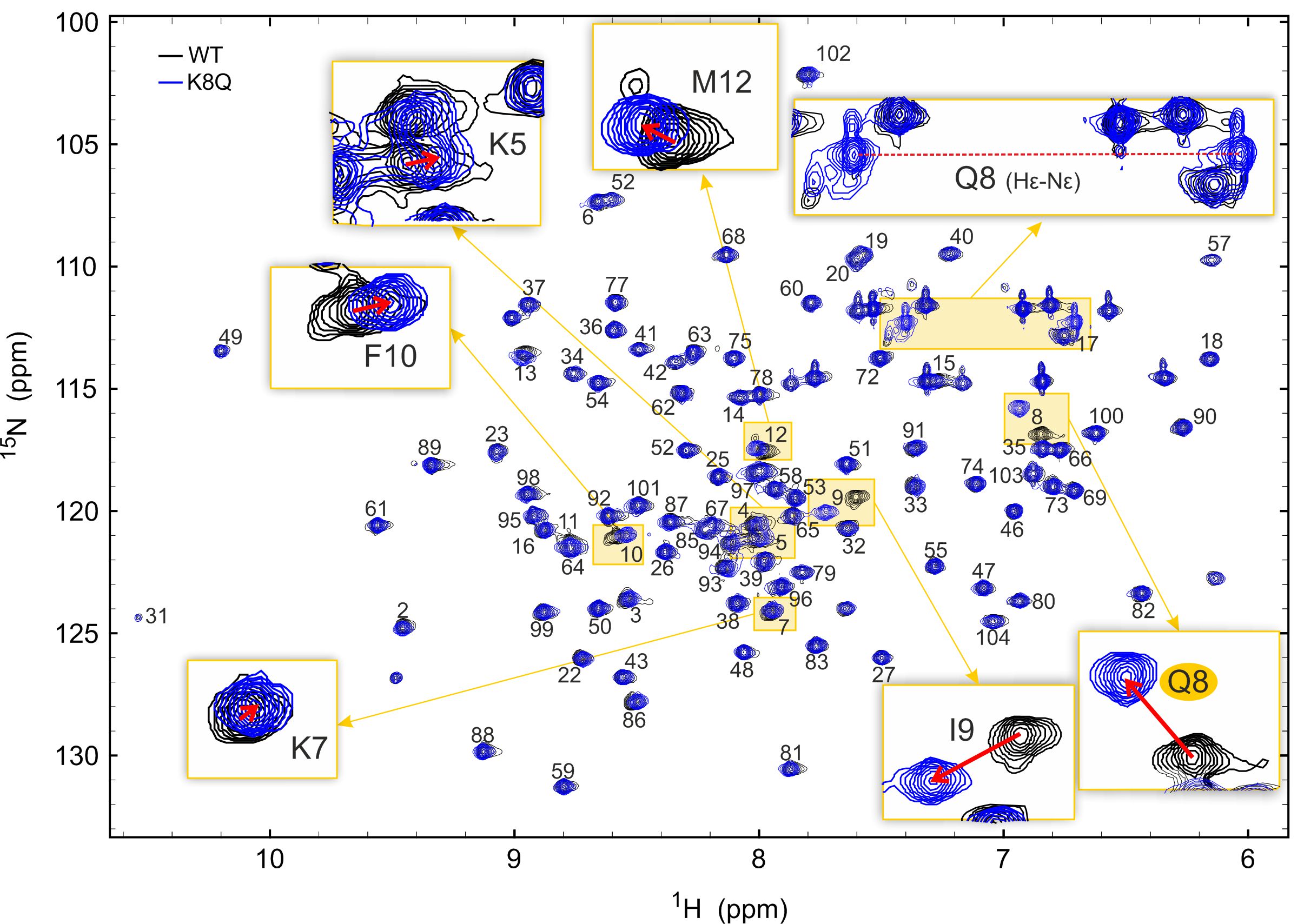


Figure S7. Superimposition of 2D ^1^H–^15^N HSQC NMR spectra of WT (black) and K8Q (blue) cytochrome *c*. *Insets*: Zooms of the signals framed in yellow boxes. The red arrows stand for the CSPs of mutant C*c* as compared with the WT species. The red dotted line shows the ^15^N chemical shift at which the ^1^H of the Q8 ε‑amine group appears. The yellow circle denotes the mutated residue.


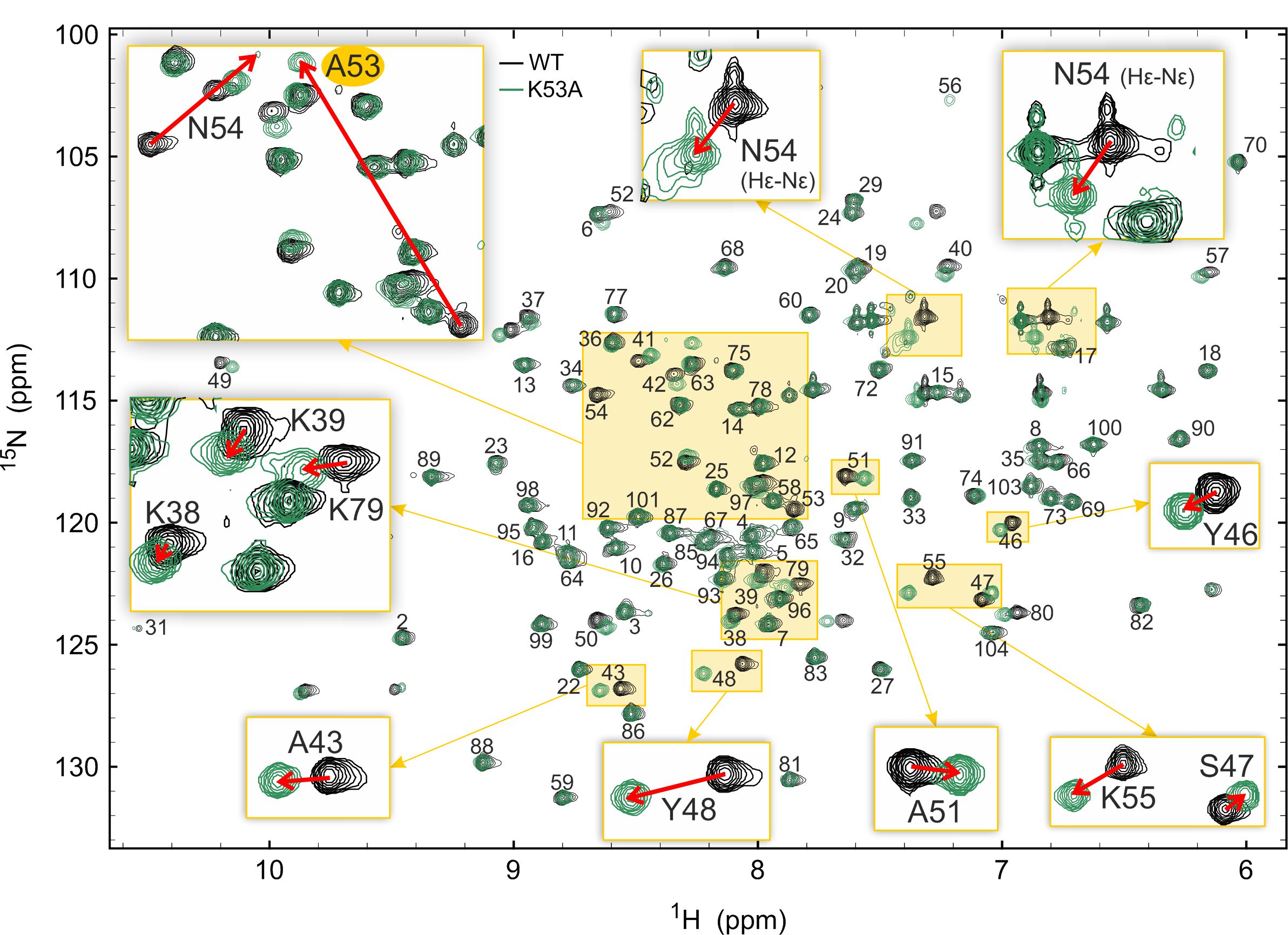


Figure S8. Superimposition of 2D ^1^H–^15^N HSQC NMR spectra of WT (black) and K53A (green) cytochrome *c*. *Insets*: Zooms of the signals framed in yellow boxes. The red arrows stands for the CSPs of mutant C*c* as compared with the WT species. The yellow circle surrounds the mutated residue.


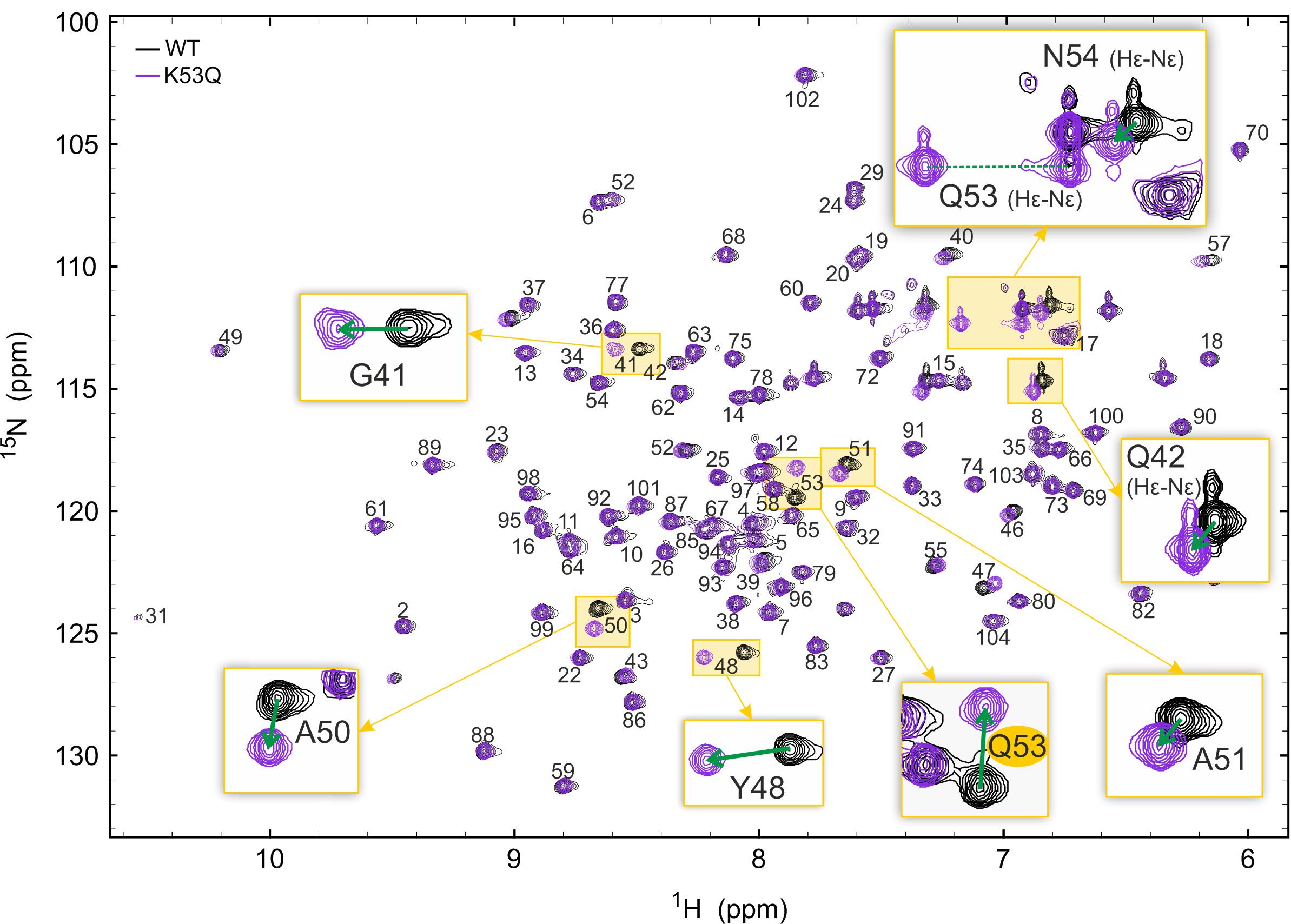


Figure S9. Superimposition of 2D ^1^H–^15^N HSQC NMR spectra of WT (black) and K53Q (purple) cytochrome *c*. *Insets*: Zooms of the signals framed in yellow boxes. The green arrows stand for the CSPs of mutant C*c* as compared with the WT species. The green dotted line shows the ^15^N chemical shift at which the ^1^H of the Q53 ε‑amine group appears. The yellow circle surrounds the mutated residue.


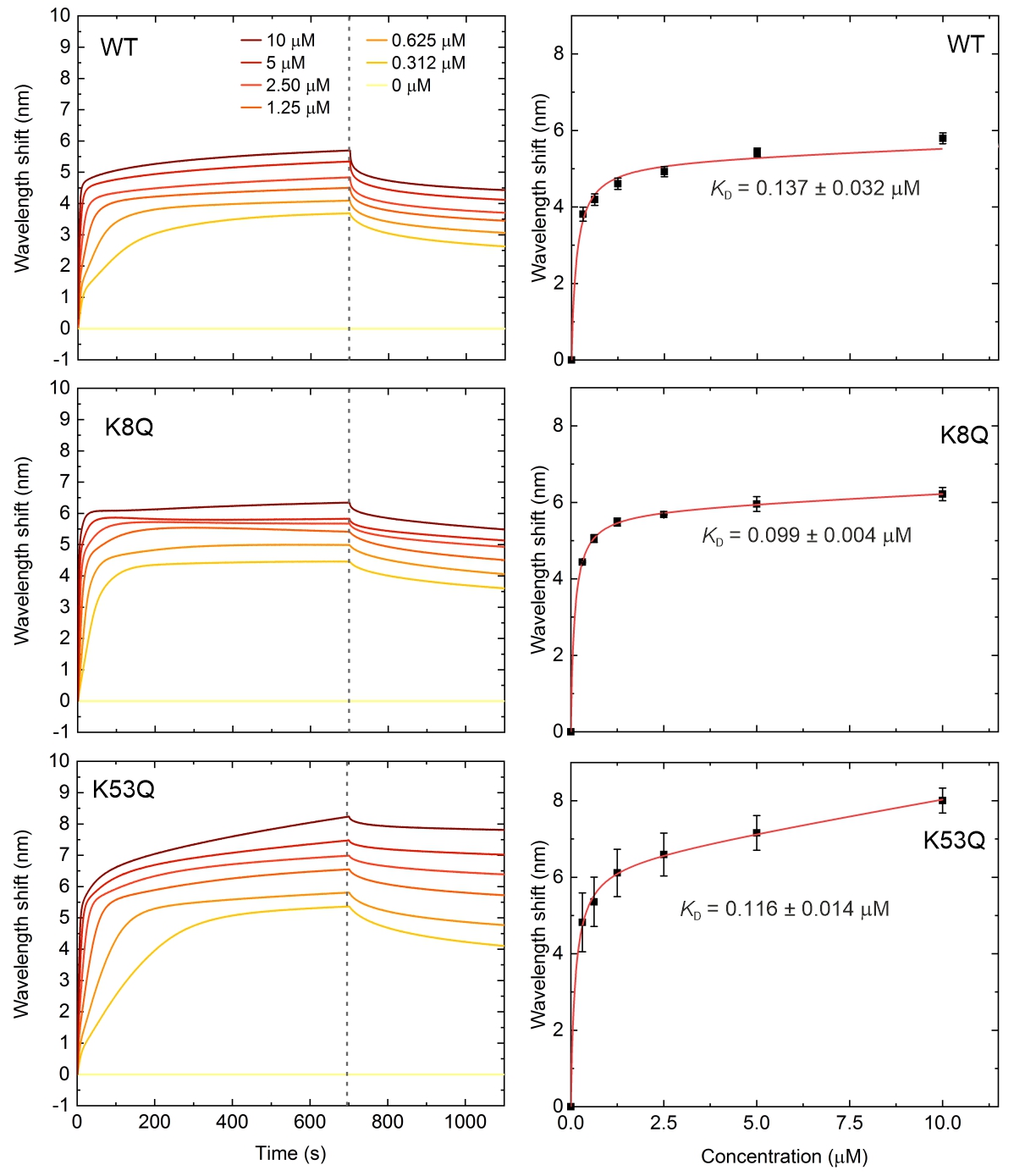


Figure S10. Interaction of WT and mutant cytochrome *c* with cytochrome *c*_1_ as determined by BLI. Wavelength shift over time (*left* panels) and calculated dissociation equilibrium constant (*K*_D_) (*right* panels) for the interaction between reduced C*c*_1_ and reduced WT, K8Q or K53Q C*c* were determined at increasing C*c* concentration. The dotted gray line in the left panels indicates the time at which the wavelength shifts were taken for calculation of the *K*_D_ values in the right panels. The data set was fitted to a non-linear equation to obtain the *K*_D_ values (red lines) (see details in Methods section). The results are expressed as the mean ± SD.


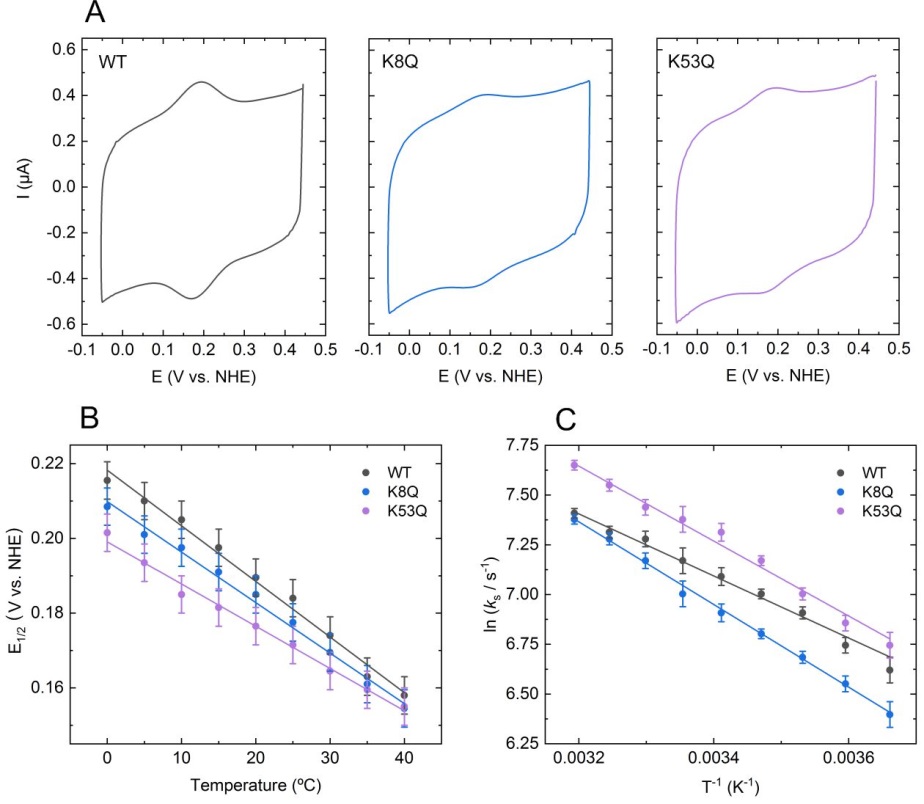


Figure S11. Electrochemical characterization of WT and mutant cytochrome *c*. (A) Cyclic voltammograms of reduced C*c* species immobilized onto a chemically modified polycrystalline gold electrode (scan rate: 0.1 V s^-1^). (B) Midpoint redox potential and (C) electron transfer rate constant for immobilized C*c* as determined at varying temperature under the same experimental conditions as in panel A. The results are expressed as the mean ± SD.


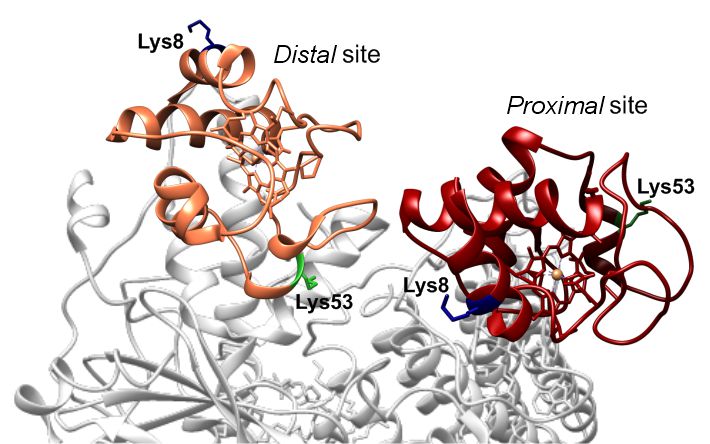


**Figure S12. Binding sites for cytochrome *c* on complex IV at the respiratory supercomplex.** The representative structure of WT C*c* at the *distal* site of complex IV is in orange, where Lys53 (green) points towards the complex IV surface (light gray), and that at the *proximal* site is in red, where Lys8 (blue) points towards the complex IV surface.

Table S1. Primers used in the design of cytochrome *c* mutants.

| **Primer** | **Sequence** |
| --- | --- |
| K8A Fwd | 5’- AGAAAGGCAAGGCGATTTTTATTATGAAGTGTTCCCAGTGC -3’ |
| K8A Rev | 5’- ATAATAAAAATCGCCTTGCCTTTCTCAACATCACCC -3’ |
| K8Q Fwd | 5’- AGAAAGGCAAGCAAATTTTTATTATGAAGTGTTCCCAGTGC -3’ |
| K8Q Rev | 5’- ATAATAAAAATTTGCTTGCCTTTCTCAACATCACCC -3’ |
| K53A Fwd | 5’- ACGGCGGCGAACGCGAACAAAGGCATCATCTGGGGCGAAG -3’ |
| K53A Rev | 5’- GATGCCTTTGTTCGCGTTCGCCGCCGTGTAGCTGTAG -3’ |
| K53Q Fwd | 5’- CGGCGGCGAACCAGAACAAAGGCATCATCTGGGGCGAAG -3’ |
| K53Q Rev | 5’- GATGCCTTTGTTCTGGTTCGCCGCCGTGTAGCTGTAG -3’ |

Table S2. Bio-Layer Interferometry protocol for cytochrome *c*_1_ – cytochrome *c* interaction.

| **Step** | **Step name** | **Time (ns)** | **Shake** | **Step type** |
| --- | --- | --- | --- | --- |
| 1 | Initial baseline | 180 | 1000 | Baseline |
| 2 | His-tagged Protein | 300 | 1000 | Loading |
| 3 | Baseline | 100 | 1000 | Baseline |
| 4 | Association | 700 | 1000 | Association |
| 5 | Dissociation | 400 | 1000 | Dissociation |
| 6 | Regeneration (10 mM glycine pH 1.7) | 5 | 1000 | Custom |
| 7 | Neutralization | 5 | 1000 | Custom |
| 8 | Regeneration (10 mM glycine pH 1.7) | 5 | 1000 | Custom |
| 9 | Neutralization | 5 | 1000 | Custom |
| 10 | Regeneration (10 mM glycine pH 1.7) | 5 | 1000 | Custom |
| 11 | Neutralization | 5 | 1000 | Custom |
| 12 | Re-charging (10 mM NiCl_2_ in H_2_O) | 60 | 1000 | Custom |

**References**

1. Sreerama N and Woody RW (2000) Estimation of protein secondary structure from circular dichroism spectra: Comparison of CONTIN, SELCON, and CDSSTR methods with an expanded reference set. *Anal Biochem* **287**, 252-260.
